# Mesenchymal stromal cells pretreated with pro‐inflammatory cytokines promote skin wound healing through VEGFC‐mediated angiogenesis

**DOI:** 10.1002/sctm.19-0241

**Published:** 2020-06-13

**Authors:** Mengting Zhu, Yunpeng Chu, Qianwen Shang, Zhiyuan Zheng, Yanan Li, Lijuan Cao, Yongjing Chen, Jianchang Cao, Oscar K. Lee, Ying Wang, Gerry Melino, Guozhong Lv, Changshun Shao, Yufang Shi

**Affiliations:** ^1^ The First Affiliated Hospital of Soochow University, State Key Laboratory of Radiation Medicine and Protection, Institutes for Translational Medicine Soochow University Medical College Suzhou People's Republic of China; ^2^ Department of Experimental Medicine and Biochemical Sciences University of Rome ‘Tor Vergata’ Rome Italy; ^3^ CAS Key Laboratory of Tissue Microenvironment and Tumor Shanghai Institute of Nutrition and Health, Shanghai Institutes for Biological Sciences Shanghai People's Republic of China; ^4^ Institute for Tissue Engineering and Regenerative Medicine The Chinese University of Hong Kong HongKong People's Republic of China; ^5^ Department of Burn Surgery The 3rd People's Hospital of Wuxi and Wuxi Medical College of Jiangnan University Wuxi People's Republic of China; ^6^ State Key Laboratory of Radiation Medicine and Protection Institutes for Translational Medicine, Soochow University Suzhou People's Republic of China

**Keywords:** angiogenesis, IFN‐γ, mesenchymal stromal cells, TNF‐α, VEGFC, wound healing

## Abstract

Skin is the largest organ of the human body. Skin wound is one of the most common forms of wound. Mesenchymal stromal cells (MSCs) have been used to aid skin wound healing via their paracrine factors. Because the secretome of MSCs can be greatly enriched and amplified by treatment with IFN‐γ and TNF‐α (IT), we here tested whether supernatant derived from MSCs pretreated with IT, designated as S‐MSCs‐IT, possesses improved wound healing effect by using a murine model of cutaneous excision, S‐MSCs‐IT was found to be more potent in promoting angiogenesis, constricting collagen deposition and accelerating wound closure than control supernatant (S‐MSCs) during the healing of skin wound. VEGFC, but not VEGFA, was greatly upregulated by IT and was found to be a key factor in mediating the improved wound healing effect of S‐MSCs‐IT. Our results indicate that the beneficial paracrine effect of MSCs on wound healing can be enhanced by pretreatment with inflammatory cytokines. IT treatment may represent a new strategy for optimizing the therapeutic effect of MSCs on skin injuries.

## Graphical abstract

TNF‐α and IFN‐γ stimulation upregulates the expression of VEGFC in mesenchymal stromal cells, which acts on endothelial cells and promotes angiogenesis, thus accelerating skin wound healing.

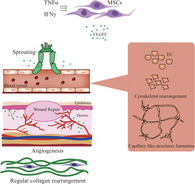

**Significance statement**

Mesenchymal stromal cells (MSCs) have been demonstrated to accelerate wound healing; however, MSCs or MSC‐conditioned medium did not exhibit superior effect in all cases, indicating that the beneficial effect of MSCs on wound healing needs to be further improved. The results of this study indicate that the beneficial paracrine effect of MSCs on wound healing can be enhanced by pretreatment with inflammatory cytokines. TNF‐α and IFN‐γ treatment may represent a new strategy for optimizing the therapeutic effect of MSCs on skin injuries.

## 1 INTRODUCTION

Skin injury is one of the most common forms of wounds.^1^ After injury, the integrity of the skin tissue must be quickly restored in order to prevent infection and minimize fluid loss.^2^, ^3^ However, the wound healing process can be compromised in many pathophysiological conditions such as diabetes, chronic renal failure, and aging. New strategies that accelerate wound healing are critically needed in clinical settings.^4^ Mesenchymal stromal cells (MSCs) are multipotent stem cells that exist in many tissues and are capable of differentiating into several different cell types.^5^ Many studies have demonstrated that intravenous or intradermal administration of MSCs dramatically enhanced cutaneous wound healing in animals and patients suffering from incisional and excisional wounds, diabetic ulcers, radiation ulcers, and burns.^6^, ^7^ It is generally believed that MSC‐based therapy does not only provide a source of cells that reconstitute tissues but also regulates inflammation. When stimulated by inflammatory cytokines, MSCs can suppress T‐cell proliferation by producing chemokines and nitric oxide (NO).^8^ MSCs were also shown to induce the differentiation of dendritic cell (DC) precursors into regulatory DCs that can alleviate bacteria‐induced liver injury.^9^ We recently reported that MSCs can produce IGF‐2 that endows maturing macrophages with anti‐inflammatory properties.^10^ Indeed, considering the limited differentiation capability of MSCs, it is hard to imagine that MSCs are capable of replacing all missing cell types during wound repair. On the other hand, optimization of tissue microenvironment for MSCs, also called “cell empowerment,” may account for much of the tissue reparation mediated by MSCs.^11^ This empowerment is likely made possible through paracrine factors. Interestingly, the MSCs‐conditioned medium has been demonstrated to accelerate wound healing.^12^ However, MSCs or MSCs‐conditioned medium did not exhibit superior effect in all cases,^13^, ^14^, ^15^ indicating that the beneficial effect of MSCs on wound healing needs to be further improved.

It has been reported that hypoxia can induce MSCs to secrete more fibroblast growth factor (FGF) and vascular endothelial growth factor (VEGF) that promote wound healing.^16^, ^17^ Our previous studies showed that activated T cells‐derived supernatant (rich in IFN‐γ and TNF‐α) can greatly stimulate the expression of a large number of secretory proteins in MSCs, with CXCL9, as an example, being upregulated over a million‐fold.^8^ Interestingly, it has been reported that in chronic pressure ulcers of bed‐restrained patients, CXCL9 expression is much lower than in wounds of healthy individuals.^18^ This low CXCL9 led to defective chemotaxis of endothelial cells (ECs) and subsequent delay in wound healing.^4^, ^19^ This suggests that acute inflammatory stimulation may endow MSCs a stronger wound healing ability.

Formation of new blood vessels, a process known as angiogenesis, is a key step for successful wound healing. Restoration of blood flow to damaged tissues provides oxygen and nutrients to support the growth and function of reparative cells.^20^ It has been reported that reduced angiogenesis is associated with delayed healing, impaired re‐epithelization, and insufficient granulation in diabetic mice.^21^ VEGF family members are believed to be the most important proangiogenic factors during wound healing.^22^, ^23^ They promote the proliferation, migration, differentiation, and survival of ECs.^24^ Studies have shown that the loss of one copy of the VEGF gene leads to abnormal blood vessel development and lethality in embryos, which highlights the importance of VEGF as a neovascularization mediator.^25^, ^26^ A member of the VEGF family, VEGFC, is necessary for the migration and survival of newly formed lymphatic ECs.^27^, ^28^, ^29^ However, the role of VEGFC in angiogenesis remains unclear.

In this study, we explored whether MSCs under acute inflammation, as mimicked by the treatment with IFN‐γ and TNF‐α (IT), could acquire a more beneficial effect on wound healing, and if so, what secretory factors mediate it. We found that the supernatant derived from MSCs pretreated with IT (S‐MSCs‐IT) could accelerate wound closure and result in a more regular collagen rearrangement. In particular, S‐MSCs‐IT could significantly enhance angiogenesis, an effect mainly mediated by the upregulation of VEGFC expression after IT stimulation. Our results not only provide an optimal MSCs‐based therapeutic strategy for wound healing but also reveal a novel role of VEGFC in angiogenesis.

## 2 RESULTS

### 2.1 Supernatant of MSCs pretreated with IT accelerates cutaneous wound closure

**FIGURE 1:**
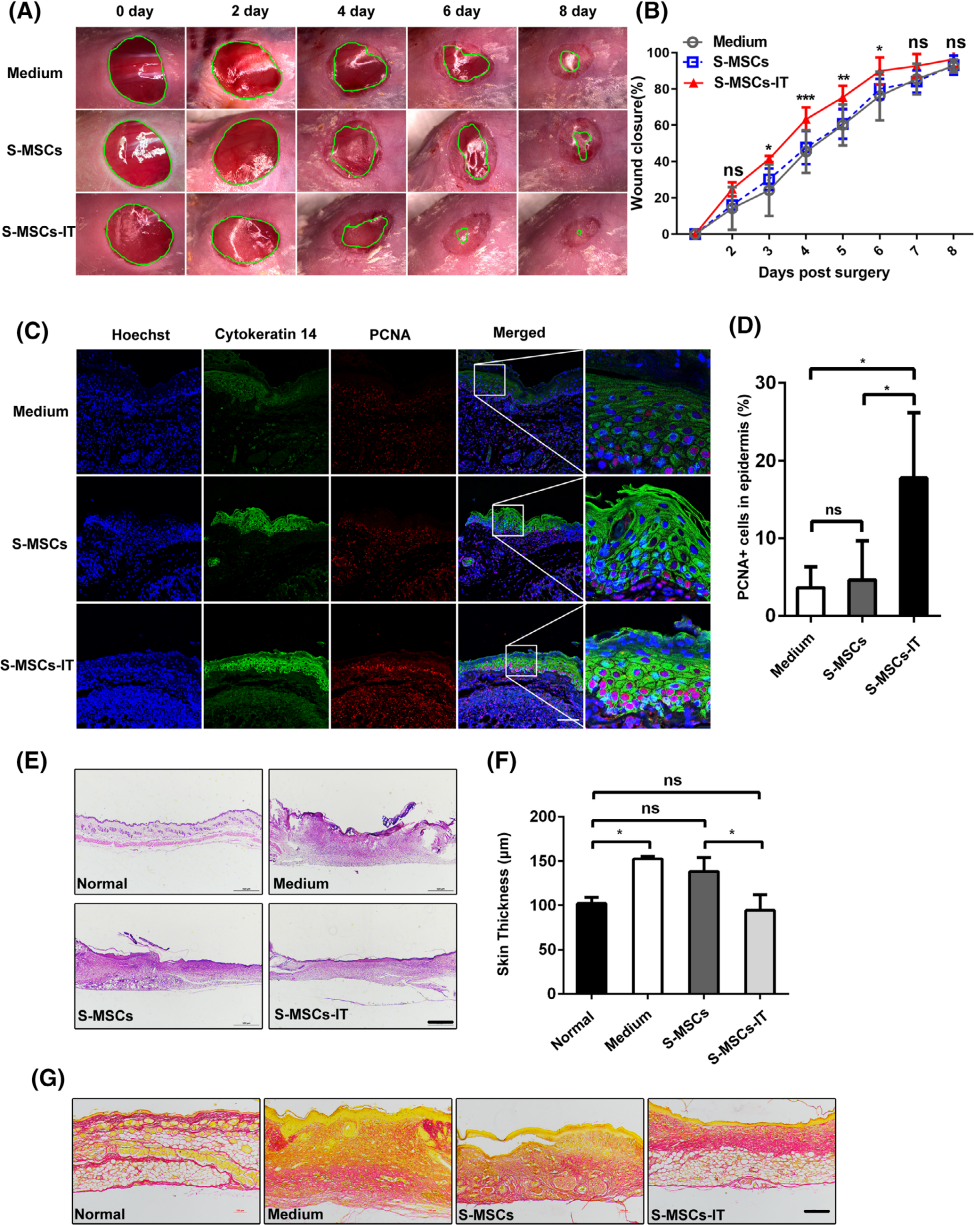
Supernatant derived from MSCs pretreated with inflammatory cytokines accelerates cutaneous wound closure and regulates collagen rearrangement. Excisional wounds were treated daily with control medium, S‐MSCs, or S‐MSCs‐IT and photographs were taken. A, Representative images of wounds are shown at the time points indicated. B, Measurements of wound sizes at different times. Significant differences were determined by one‐wayanalysis of variance, *P < .05, **P < .01, ***P < .001 (*n* = 4‐5 mice for each group and time point). C, Cutaneous wounds on day 6 post of injury were stained with PCNA and Cytokeratin 14 and micrographs were taken. Scale bar = 50 μm. D, Quantification of the number of proliferating cell nuclear antigen (PCNA)‐positive keratinocytes. Significant differences were determined by one‐way analysis of variance, *P < .05 (*n* = 4‐5 mice for each group). E,F, Wound histology after H&E (scale bar = 500 μm) and measurement of skin thickness. Significant differences were determined by one‐way analysis of variance, *P < .05 (*n* = 4‐5 mice for each group). G, Sirius red staining (scale bar = 200 μm). Tissue sections obtained from the wound areas on day 10 post of injury were stained with H&E and Sirius red. Representative micrograph images are shown. The MSCs are from the human umbilical cord (defined as SD4). All of the data are representative of two independent experiments. Data are shown as mean ± SD. MSCs, mesenchymal stromal cells

It has been shown that MSCs could promote wound healing via their paracrine factors. To determine whether the beneficial effect of MSCs on wound healing can be enhanced by pretreatment with IT, we examined the effects of supernatant derived from human umbilical cord‐derived MSCs (UC‐MSCs) with and without pretreatment with IT on skin regeneration using the excisional cutaneous wound‐healing mouse model.^16^ The MSCs used in in this study were phenotypically characterized using a set of surface markers and identified for their differentiation ability of adipogenesis and osteogenesis, the doubling time is about 20 hours (Figure S1A‐D). MSCs were stimulated with IT (20 ng/mL for each cytokine) when they reached 90% confluence, 24 hours later, MSCs were washed with phosphate‐buffered solution (PBS) and then cultured in fetal calf serum (FBS)‐free medium for 12 hours to collect the supernatant. The MSCs supernatant or control medium (20 μL per wound) were applied topically onto the skin wounds once daily for 8 days. Compared with control medium or control MSC supernatant (S‐MSCs), supernatant from MSCs treated with IT (S‐MSCs‐IT) accelerated wound closure. Whereas wound areas were time‐dependently decreased in all experimental groups, the reduction in wound areas of S‐MSCs‐IT treated group was more pronounced starting from day 3 (Figure 1A,B). The supernatant derived from human adipose‐derived MSCs treated with IT also promoted wound closure although the effect was less pronounced than that of UC‐MSCs (Figure S2A,B). The proliferation and migration of keratinocytes are associated with re‐epithelialization.^30^ We further evaluated the effect of S‐MSCs‐IT on wound healing by evaluating the proliferation of keratinocytes. Keratinocytes exhibited a higher proliferation rate in wounds treated with S‐MSCs‐IT than those treated with S‐MSCs (Figure 1C,D). On day 10, the wounds were completely covered by new skins in all treated groups, but the skin thickness and histology in S‐MSCs‐IT group more resembled those in normal skin (Figure 1E,F). Since collagen deposition accompanies wound healing,^31^ we performed Sirius red staining of skin wounds and found that collagen was more regularly arranged and more constricted in S‐MSCs‐IT treated skins than that in those treated with control medium and S‐MSCs (Figure 1G), These results indicate that S‐MSCs‐IT does not only accelerate wound closure but also promote collagen constriction.

### 2.2 Supernatant derived from MSCs pretreated with IT accelerates angiogenesis

**FIGURE 2:**
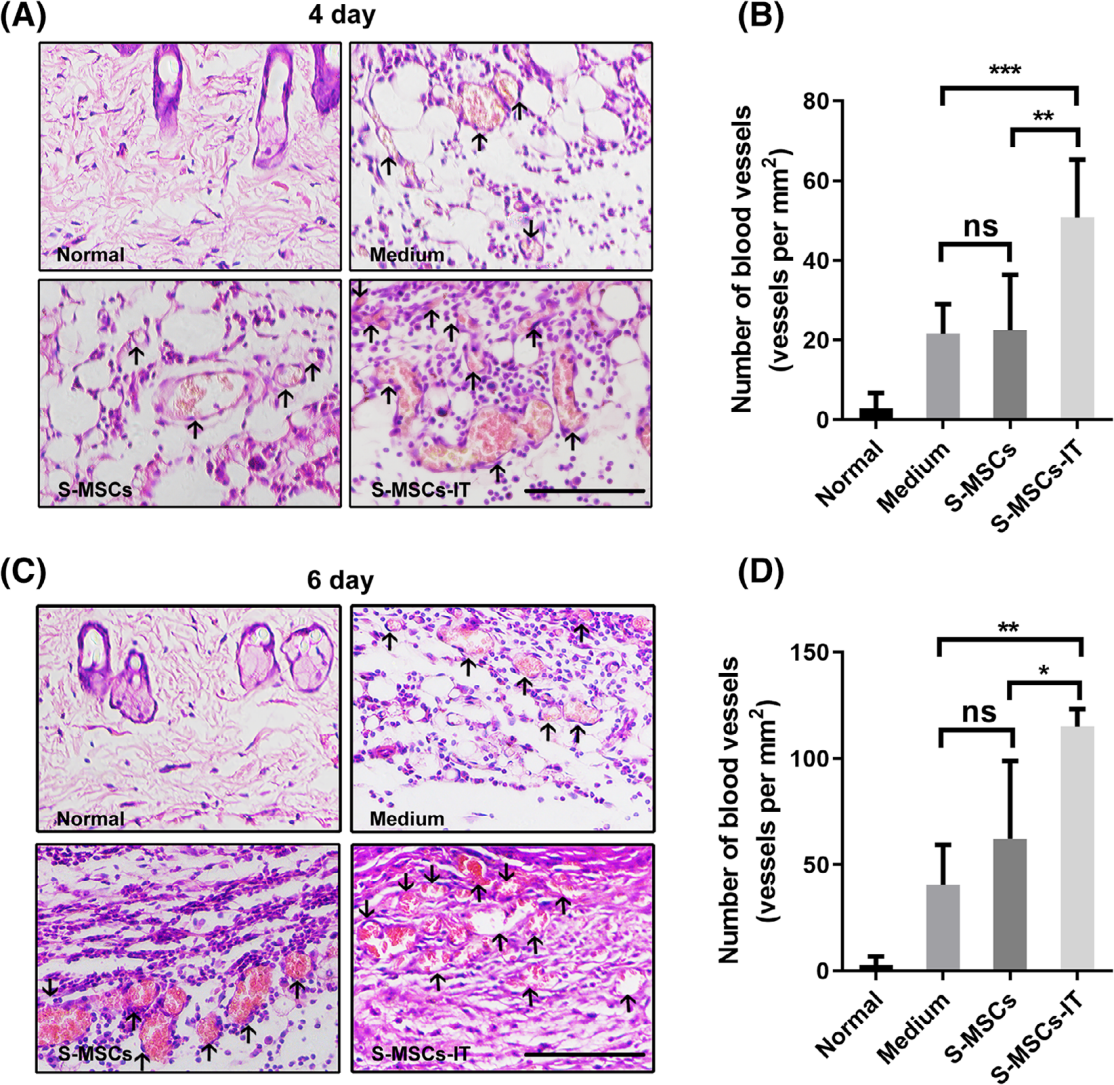
Supernatant derived from MSCs pretreated with inflammatory cytokines promotes angiogenesis. Cutaneous wounds on, A,B, day 4 and, C,D, day 6. A,C, Cutaneous wounds were stained with H&E and micrographs were taken. Black arrows indicate blood vessels containing red blood cells. Scale bar = 100 μm. B,D, The number of blood vessels containing red blood cells in each section was counted at the indicated time points. Results are presented as the number of blood vessels per mm^2^. Significant differences were determined by one‐way analysis of variance, *P < .05, **P < .01, ***P < .001 (*n* = 4‐5 mice for each group). The MSCs are from the human umbilical cord (defined as SD4). Data are representative of two independent experiments. Data are shown as mean ± SD. MSCs, mesenchymal stromal cells

Angiogenesis is critically required for wound healing.^20^ To evaluate the role of S‐MSCs‐IT in angiogenesis, we scored the number of blood vessels in tissue sections of cutaneous wounds after treatment with control medium, S‐MSCs, and S‐MSCs‐IT respectively, from day 4 to day 6. The number of blood vessels containing red blood cells was higher in S‐MSCs‐IT treated wounds than in control medium or S‐MSCs treated wounds (Figure 2A,B, *P* < .01, *P* < .001; Figure 2C,D, *P* < .05, *P* < .01; Figure S3A‐D), indicating enhanced angiogenesis by S‐MSCs‐IT during wound repair. CD31 immunohistochemical staining results confirmed the increased angiogenesis (Figure S3E‐H). Together, these results demonstrated that S‐MSCs‐IT possesses a potent angiogenic effect during cutaneous wound healing.

### 2.3 VEGFC mediates the pro‐angiogenic ability of S‐MSCs‐IT by promoting cytoskeleton rearrangement in ECs

**FIGURE 3:**
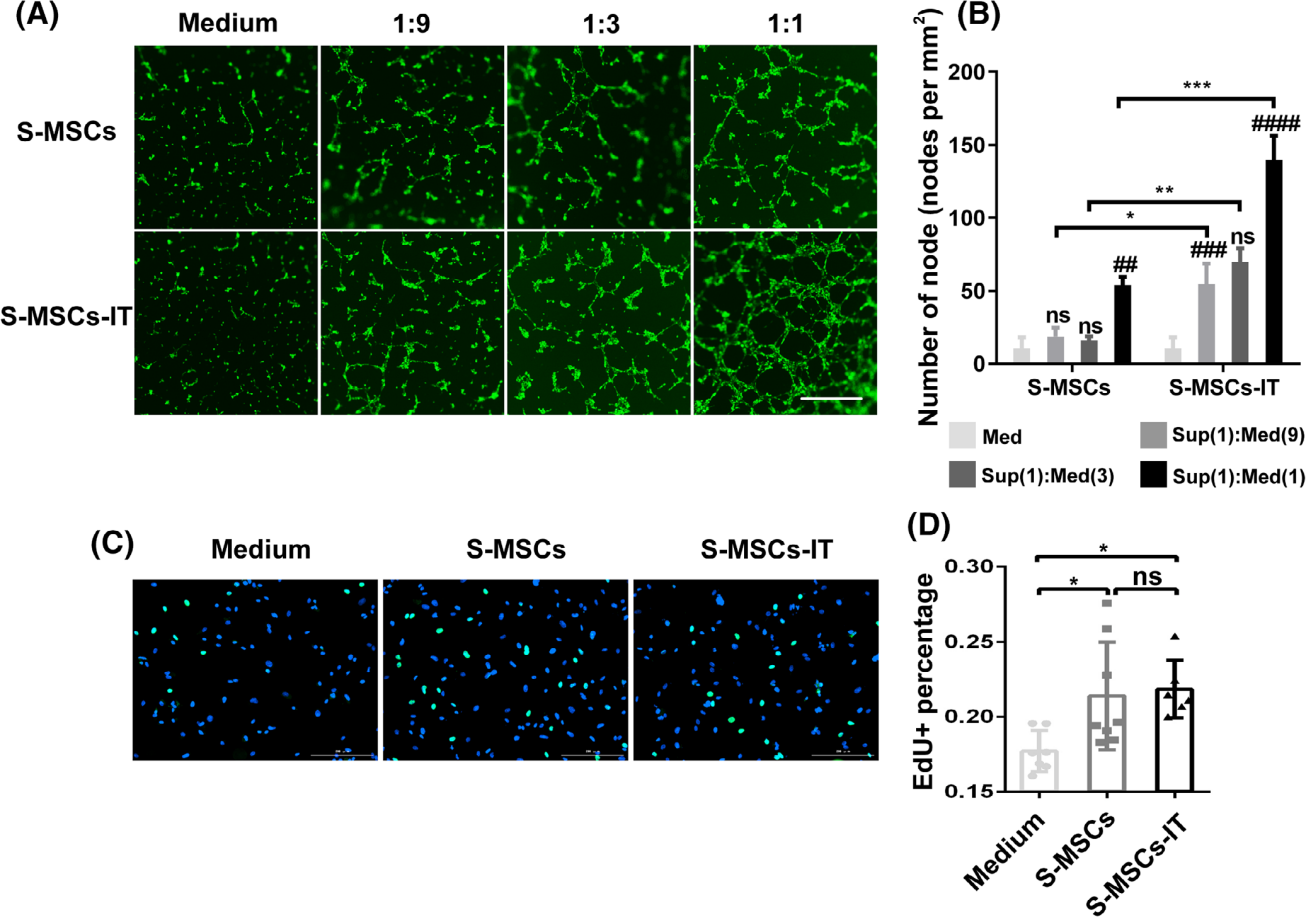
Supernatant derived from MSCs pretreated with inflammatory cytokines promotes the formation of capillary‐like structures by endothelial cells in vitro. A, Tube‐formation assay: representative images showing tube formation of green fluorescent protein (GFP)‐transgenic HUVECs cultured in control medium, S‐MSCs, or S‐MSCs‐IT on Matrigel for 6 hours. Scale bar = 500 μm. B, Quantitative analysis of the number of nodes. Differences were determined by one‐way analysis of variance, *P < .05, **P < .01, ***P < .001, ^*#*^ *P* < .05, ^*##*^ *P* < .01, ^*###*^ *P* < .001, ^*####*^ *P* < .0001. *#* represents the comparison to the previous one within groups. Data are shown of three replicates and representative of two independent experiments. C, Representative images showing proliferation of HUVECs cultured in control medium, S‐MSCs, or S‐MSCs‐IT for 6 hours. D, Analysis of the proliferation of HUVECs. Differences were determined by one‐way analysis of variance, *P < .05. Data are representative of three independent experiments. The MSCs are from the human umbilical cord (defined as SD4). Data are shown as mean ± SD. HUVEC, human umbilical‐derived vein endothelial cell; MSC, mesenchymal stromal cell

To determine the paracrine effect of IT‐stimulated MSCs on angiogenesis in vitro, we cultured 3 × 10^4^ green fluorescent protein (GFP)‐transduced human umbilical‐derived vein endothelial cells (HUVECs) on Matrigel with regular medium, S‐MSCs, and S‐MSCs‐IT, respectively. Scoring of tubular branches indicated that the formation of capillary‐like structures was slightly enhanced by S‐MSCs compared with medium, but the effect of S‐MSCs‐IT, in a concentration‐dependent manner, was more pronounced (Figure 3A,B). The ability of TNF‐α and IFN‐γ to augment the paracrine angiogenic effect of MSCs was confirmed using a separate UC‐MSC line (Figure S4A,B). The proangiogenic effect of adipose‐derived stromal cells (ADSCs), however, appeared to be only slightly increased by TNF‐α and IFN‐γ treatment (Figure S5A,B). We also evaluated the proliferation of HUVECs using the EdU incorporation assay but detected no difference between S‐MSCs and S‐MSCs‐IT groups (Figure 3C,D, *P* > .05), suggesting that S‐MSCs‐IT does not promote angiogenesis by further stimulating the proliferation of ECs when compared to S‐MSCs.

**FIGURE 4:**
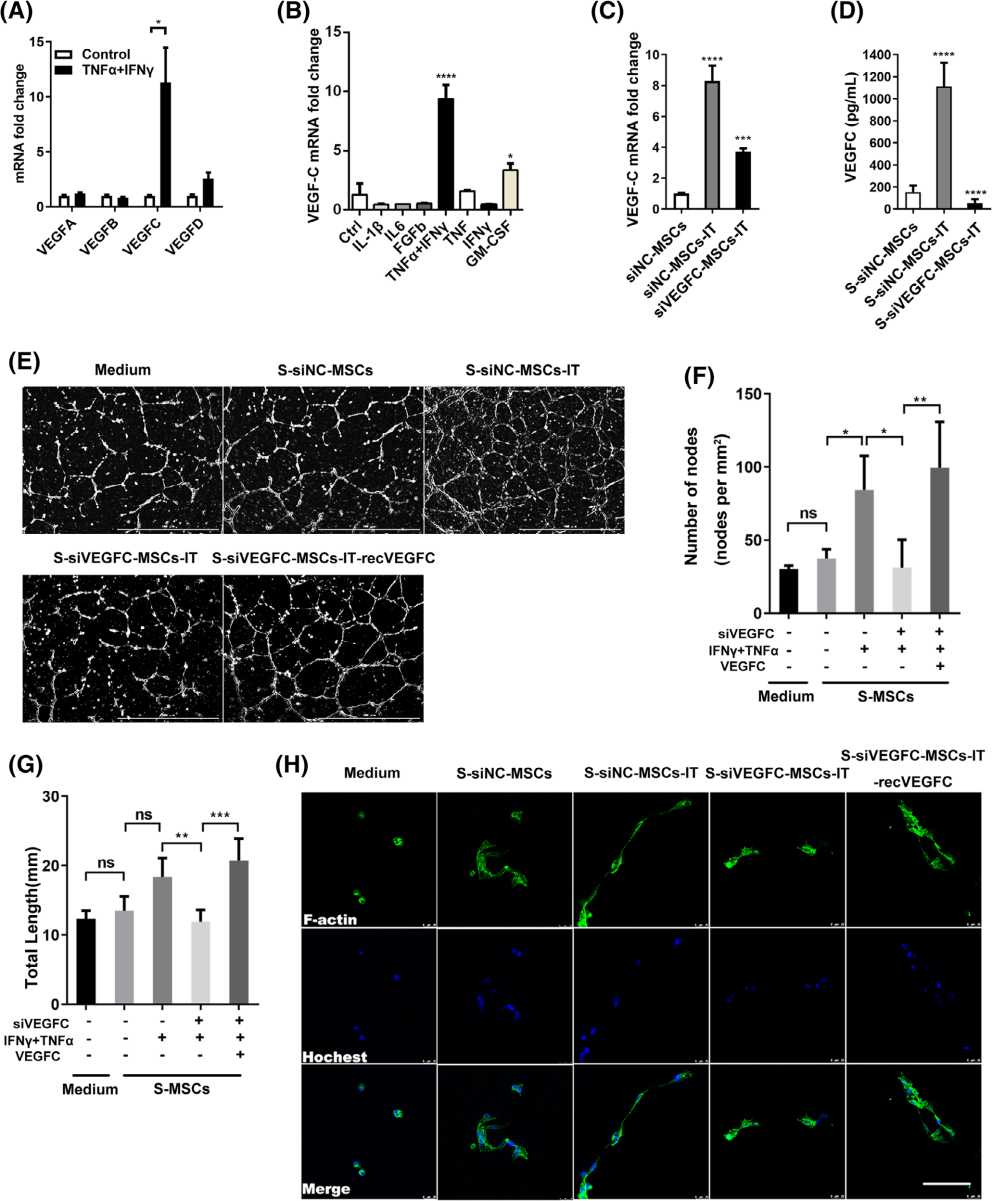
VEGFC (vascular endothelial growth factor C) mediates the pro‐angiogenic ability of S‐MSCs‐IT by regulating the cytoskeleton rearrangement of HUVECs. A, The mRNA expression of VEGF‐A, B, C, D after IFN‐γ and TNF‐α stimulation for 24 hours. Differences were determined by unpaired *t* test, *P < .05. Data are representative of three independent experiments. B, The mRNA expression of VEGFC in MSCs after different cytokines stimulation for 24 hours. C, The mRNA expression of VEGFC in MSCs and, D, the protein concentration in MSC culture supernatant after VEGFC knockdown. Significance was determined by one‐way analysis of variance, *P < .05, **P < .01, ***P < .001, ****P < .0001. Data are representative of three independent experiments. E,H, Representative images showing tube‐formation and cytoskeleton rearrangement of HUVECs cultured in S‐siNC‐MSCs, S‐siNC‐MSCs‐IT, S‐siVEGFC ‐MSCs‐IT, or S‐siVEGFC‐MSCs‐IT‐recVEGFC; scale bar = 1000 μm for tube formation and scale bar = 25 μm for cytoskeleton rearrangement. F,G, Quantitative analysis of the number of nodes and total length. Differences were determined by one‐way analysis of variance, *P < .05, **P < .01, ***P < .001. Data are representative of three independent experiments in (E), two independent experiments in (H). The MSCs are from the human umbilical cord (defined as SD4). Data are shown as mean ± SD. HUVEC, human umbilical‐derived vein endothelial cell; MSC, mesenchymal stromal cell

We previously reported that a large number of secretory proteins were upregulated in MSCs when stimulated with activated T‐cell supernatant, which is rich in IFN‐γ and TNF‐α.^8^ As VEGF family members are the most important proangiogenic factors during wound healing, we next determined their expressions, by quantitative polymerase chain reaction (PCR), in MSCs after stimulation by IT for 24 hours. Interestingly, the results showed that VEGFC, but not the other VEGF family members, was greatly upregulated (Figure 4A). Granulocyte‐macrophage clony stimulating factor (GM‐CSF) could also stimulate the expression of VEGFC, but to a much lesser extent than IT (Figure 4B). The expression of VEGFC in ADSCs was also increased after IT stimulation (Figure S6A,B). To explore whether VEGFC mediated the increased proangiogenic effect of S‐MSCs‐IT, we knocked down VEGFC in MSCs with siRNA (Figure 4C,D). Supernatant derived from si‐VEGFC MSCs pretreated with IT (S‐siVEGFC‐MSCs‐IT) no longer possessed the angiogenic effect (Figure 4E‐G; Figure 4F, *P* < .05; Figure 4G, *P* < .01). However, supplementation of recombinant VEGFC (2.5 ng/mL) could restore the angiogenic effect of S‐siVEGFC‐MSCs‐IT in vitro (Figure 4E‐G; Figure 4F, *P* < .01; Figure 4G, *P* < .001). A previous study indicated that cytoskeleton rearrangement is necessary for the initiation of de novo lumen formation^32^; therefore, we cultured HUVECs on Matrigel with S‐MSCs‐IT for 2 hours and examined the immunofluorescence staining of F‐actin. We observed a more elongated layout of the cytoskeleton in ECs treated with S‐siNC‐MSCs‐IT than with S‐siVEGFC‐MSCs‐IT. Moreover, the exogenous VEGFC protein (2.5 ng/mL) can rescue cytoskeleton elongation (Figure 4H). These results indicate that IT could stimulate MSCs to produce more VEGFC that functions to promote vessel formation by ECs. However, proliferation of HUVECs could not be enhanced by the exogenous VEGFC (Figure S7A), which is consistent with the results obtained with S‐MSCs‐IT (Figure 3C,D). While VEGFC was reported to possess protective effect on lymphatic ECs,^33^ VEGFC did not exhibit protective effect on HUVECs exposed to H_2_O_2_ (Figure S7B), suggesting that VEGFC may function differently in different types of ECs.

### 2.4 VEGFC promotes wound closure

**FIGURE 5:**
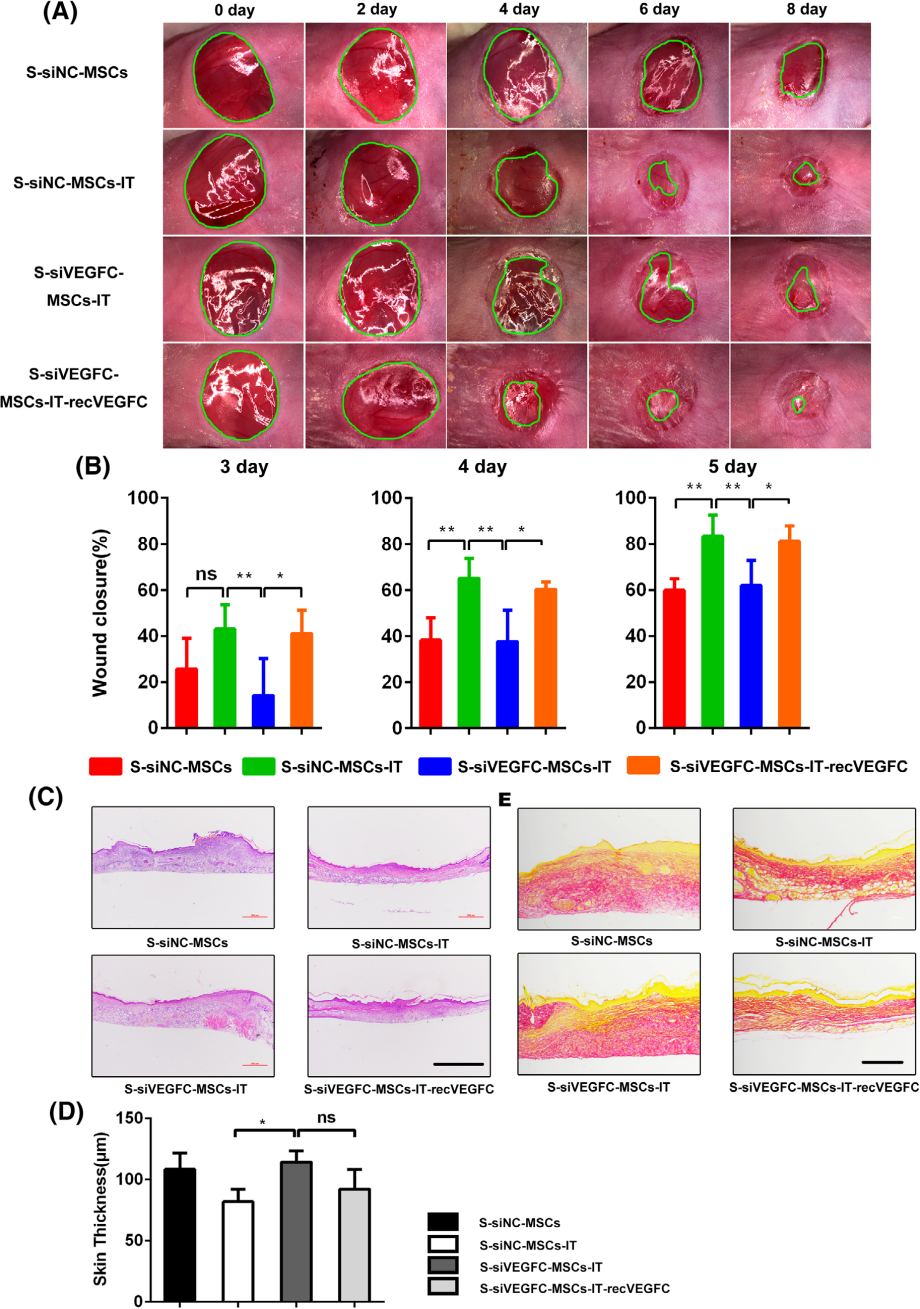
VEGFC (vascular endothelial growth factor C) accelerates cutaneous wound closure and regulates collagen rearrangement. Excisional wounds were treated daily with S‐siNC‐MSCs, S‐siNC‐MSCs‐IT, S‐siVEGFC‐MSCs‐IT, or S‐siVEGFC‐MSCs‐IT‐recVEGFC, respectively, and photographs were taken. A, Representative photographs of wounds are shown at the time point indicated. B, Measurements of wound sizes at different time points post of injury. Areas of the wounds were determined by quantitative analysis of wound images using ImageJ software. Differences were determined by one‐way analysis of variance, *P < .05, **P < .01 (*n* = 4‐6 mice for each group and time point). C,D, Wound histology after H&E (scale bar = 500 μm) and measurement of skin thickness. Significant differences were determined by one‐way analysis of variance, *P < .05 (*n* = 4‐6 mice for each group). E, Sirius red staining (scale bar = 200 μm). Tissue sections obtained from the wound area at day 10 post of injury were stained with H&E and Sirius red. Representative micrographs images are shown. The MSCs are from the human umbilical cord (defined as SD4). All of the data are representative of two independent experiments. Data are shown as mean ± SD. MSC, mesenchymal stromal cell

Next, to evaluate the role of VEGFC in S‐MSCs‐IT stimulated wound closure, we depleted VEGFC in MSCs with siRNA before pro‐inflammatory factor stimulation, then collected the supernatant to treat the excisional cutaneous wounds. We found that the enhanced ability of S‐MSCs‐IT to promote wound closure was lost upon VEGFC depletion but was restored after the addition of exogenous recombinant VEGFC protein. The rate of wound closure in S‐siVEGFC‐MSCs‐IT treatment group was significantly decreased from day 3 to day 5 when compared to that in S‐siNC‐MSCs‐IT group. However, when recombinant VEGFC protein was added into S‐siVEGFC‐MSCs‐IT, the wound closure rate was comparable to that in the S‐siNC‐MSCs‐IT group (Figure 5A,B, day 3, *P* = .0057; at day 4, *P* = .0008; at day 5, *P* = .0006). Immunofluorescence staining also revealed a reduced rate of keratinocyte proliferation in S‐siVEGFC‐MSCs‐IT treated wounds, while S‐siVEGFC‐MSCs‐IT‐recVEGFC treated wounds showed higher keratinocytes proliferation activity as S‐siNC‐MSCs‐IT treated skin (Figure S8A,B, *P* < .05, *P* > .05). Therefore, VEGFC is a key factor in mediating the acceleration of wound healing by the secretome of IT‐stimulated MSCs. We also evaluated the effect of VEGFC in S‐MSCs‐IT on skin thickness and collagen deposition. The collagen in S‐siVEGFC‐MSCs‐IT treated wounds appeared irregular and more scattered, and the newly formed skins were thicker when compared to S‐siNC‐MSCs‐IT. However, both abnormalities could be rescued by the supplementation of recombinant VEGFC protein (Figure 5C‐E). Interestingly, it was reported that VEGFC could increases collagen constriction in vitro.^34^ However, how VEGFC regulates the collagen rearrangement during skin wound healing remains to be determined.

### 2.5 VEGFC promotes angiogenesis in vivo

**FIGURE 6:**
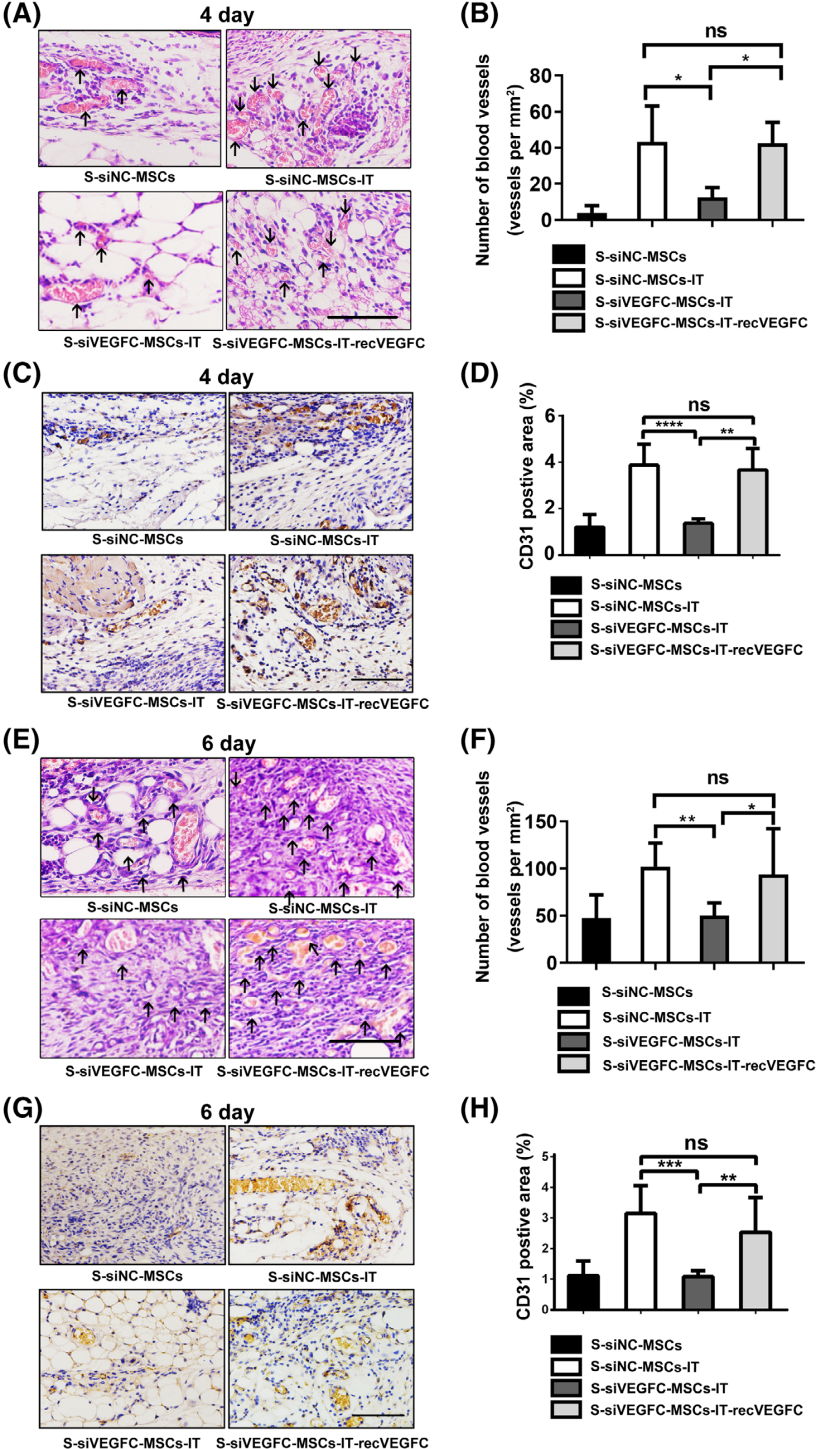
VEGFC (vascular endothelial growth factor C) promotes angiogenesis. Cutaneous wounds on, A‐D, day 4 and, E‐H, day 6. A,E, Cutaneous wounds were stained with H&E and micrographs were taken. Black arrows indicate blood vessels containing red blood cells. Scale bar = 100 μm. B,F, The number of blood vessels containing red blood cells in each section was counted at the indicated time points. Results are presented as the number of blood vessels per mm^2^. Statistical significance was determined by one‐way analysis of variance, *P < .05, **P < .01 (*n* = 4‐6 mice for each group). Data are representative of two independent experiments. C,G, Representative photographs showing CD31 immunohistochemistry in cutaneous wounds. Scale bar = 100 μm. D,H, Graph of CD31immunohistochemistry in these groups. Results are presented as the CD31 positive area (%). Significant differences were determined by one‐way analysis of variance, **P < .01, ***P < .001, ****P < .0001 (*n* = 4‐6 mice for each group). Data are one independent experiment. The MSCs are from the human umbilical cord (defined as SD4). Data are shown as mean ± SD. MSC, mesenchymal stromal cell

To determine whether the acceleration of wound closure by S‐MSCs‐IT is mediated by the pro‐angiogenic ability of VEGFC, we topically applied S‐siVEGFC‐MSCs‐IT onto the wounds and scored the newly formed blood vessels in tissue section. The results showed that the number of blood vessels in the S‐siVEGFC‐MSCs‐IT treatment group was significantly lower than that in the S‐siNC‐MSCs‐IT group after excision, but when the recombinant VEGFC protein was added to S‐siVEGFC‐MSCs‐IT, the pro‐angiogenic effect was well restored (Figure 6A,B, *P* < .05, *P* < .05; Figure 6C,D, *P* < .0001, *P* < .01; Figure 6 E,F, *P* < .01, *P* < .05; Figure 6G,H, *P* < .001, *P* < .01; Figure S9A‐D). These results indicated that S‐MSCs‐IT can promote angiogenesis during skin wound repair through VEGFC. However, how VEGFC promotes angiogenesis needs to be further investigated.

## 3 DISCUSSION

Wound healing is essential for the maintenance of body integrity and the prevention of infections. The process of acute wound healing is usually divided into three overlapping phases known as inflammation, proliferation, and remodeling.^1^ Inflammation in response to tissue damage not only eliminates necrotic cells and resists microbial infection but also is indispensable for the initiation of tissue repair.^35^ To explore the influence of inflammation on the therapeutic activity of MSCs, we subjected MSCs in culture to inflammatory cytokines and then collected the supernatant from the treated MSCs and applied it to mouse skin wounds. Our results indicate that upon stimulation by IFN‐γ and TNF‐α MSCs become more potent in promoting wound healing, an effect that is mediated by the upregulation of VEGFC.

Inflammation can not only recruit MSCs to injured tissue but also stimulate them to produce a variety of cytokines and chemokines, which in turn quell inflammation and promote repair and regeneration of the damaged tissue.^5^, ^8^, ^36^ MSCs expanded in vitro have been widely tested in wound healing studies.^6^, ^37^, ^38^, ^39^ Because the paracrine factors produced by MSCs play a critical role in wound repair, we speculated that stimulation of MSCs with inflammatory factors in vitro could enable MSCs to achieve a more beneficial effect on tissue repair. Indeed, we observed that the culture supernatant of MSCs prestimulated by IT performed better on skin wound healing than that of those without IT stimulation (Figure 1A,B). S‐MSCs‐IT treatment resulted in a more condensed and regular collagen rearrangement. More importantly, it is more potent in promoting angiogenesis that is critically required for tissue repair. Impaired angiogenesis often leads to delayed or unsuccessful wound healing. Many studies have shown that MSCs, as well as their culture supernatant can promote angiogenesis, and VEGFA, which is abundantly produced by MSCs, is believed to mediate such an effect.^40^, ^41^ Our results showed that S‐MSCs‐IT can further enhance the tubule‐forming ability of ECs when compared to S‐MSCs (Figure 3A,B). Interestingly, IT pretreatment greatly increased the expression of VEGFC, but not that of VEGFA in MSCs (Figure 4A). Depletion of VEGFC in MSCs by RNAi could abolish the effect endowed by IT on angiogenesis and wound healing, indicating that S‐MSCs‐IT could further promote angiogenesis through increased production of VEGFC (Figures 5A,B and 6A‐H).

VEGFC is believed to promote the migration and proliferation of lymphatic ECs mainly through its binding to VEGFR3.^28^ However, the receptors for VEGFC include not only VEGFR3 but also VEGFR2, though the latter mainly binds to VEGFA.^42^ This suggests that VEGFC may also bind to VEGFR2 and thereby promote angiogenesis. It has been reported that vascular sprouting relies on the coordinated activity of migrating tip cells at the forefront and proliferating stalk cells that elongate the sprout, prominent is the activation of VEGFR signaling in tip cells, which induce these cells to extend numerous filopodia,^43^ this is consistent with the elongated layout of the cytoskeleton in ECs observed in our study. When the recombinant VEGFC protein was added to S‐siVEGFC‐MSCs‐IT, the pro‐angiogenic effect was well restored (Figure 6A‐H). Our study thus supports a role of VEGFC in promoting angiogenesis and wound healing, likely through direct effects on ECs. Furthermore, we also observed that on the sixth day after the injury, keratinocytes in the S‐siNC‐MSCs‐IT treatment group proliferated faster, while in the S‐siVEGFC‐MSCs‐IT treatment group became slower. Perhaps the proliferation of keratinocytes is another contributor to wound healing, which is indirectly caused by VEGFC promoting angiogenesis to provide oxygen and nutrients, but it is also possible that VEGFC directly promotes proliferation of keratinocytes through binding to VEGFR2 expressed on keratinocytes.^44^ It will be important to further explore the expression and function of VEGFC in wound healing.

In summary, our results show that the therapeutic effect of MSCs on wound healing can be further enhanced by prestimulation with IFN‐γ and TNF‐α, via the upregulation of VEGFC. Studies aimed at optimizing the combination of different inflammatory factors may further increase the beneficial effect of MSCs on tissue repair.

## 4 MATERIALS AND METHODS

### 4.1 Materials

#### 4.1.1 *Mesenchymal stromal cells*

##### Human UC‐MSCs

The MSCs used in our experiments were isolated from human umbilical cords as previously described.^45^ Briefly, with parental consent, umbilical cords from healthy and full‐term delivery were obtained, and were transferred immediately to the lab in sterile normal saline solution within 2 hours, containing 200 mg/mL penicillin and streptomycin. Gelatinous tissues without blood vessels were separated from the umbilical cord in a PBS and cut into small pieces, which were then transferred to 10 cm diameter dishes and covered by Dulbecco's Modified Eagle Medium (DMEM) (SH30021.01, HyClone) supplemented with 10% FBS (03.A16001DC, EallBio), 2 mM glutamine, 100 mg/mL penicillin and streptomycin (15 140 163, Thermo Fisher Scientific) at 37°C under 5% CO_2_. Nonadherent cells were removed after 24 hours, and adherent cells were maintained with medium replenishment every 3 days.

#### 4.1.2 *Endothelial cells*

Primary HUVECs were purchased from PromoCell (C‐12203). Cells were cultured in Endothelial Cell Growth Medium with SupplementMix (C‐22010, PromoCell, Germany), and were used at passages from 3 to 5.

### 4.2 Methods

#### 4.2.1 *Preparation of conditioned medium*

MSCs were cultured in 100‐mm diameter culture dishes until they reached 90% confluence, then were stimulated with IT (20 ng/mL IFN‐γ and TNF‐α, NBP2‐34992‐100ug/NBP2‐35076‐100ug, NOVUS), 24 hours later, MSCs were washed with PBS to remove the cytokines and then cultured in FBS‐free medium for 12 hours. Conditioned medium was collected and centrifuged at 300*g* for 5 minutes to remove cell debris. MSCs were transfected with VEGFC‐specific small interfering RNAs (sense 5′‐3′ GCA AAG AUC UGG AGG AGC AdTdT, antisense 5′‐3′ UGC UCC UCC AGA UCU UUG CdTdT). The transfections were performed using the interferin transfection reagent (PT‐409‐10, Polyplus Transfection, France) according to the manufacturer's protocol. Nonsilencing siRNA (sense 5′‐3′UUU UCC GAA CGU GUC ACG UdTdT, anti‐sense 5′‐3′ACG UGA CAC GUU CGG AGA AdTdT) was used to control for any effect of siRNA and the transfection reagent. After 24 hours, MSCs were washed with PBS and cultured in a new complete medium and stimulated with 20 ng/mL IFN‐γ and TNF‐α for 24 hours. The cells were then washed with PBS and cultured in FBS‐free medium for 12 hours, conditioned medium was centrifuged at 300*g* for 5 minutes to remove cell debris and stored at −80°C for the following experiments.

#### 4.2.2 *Cell culture and lentiviral transfection*

An amount of 10 × 10^4^ human umbilical‐derived vein endothelial cells (HUVECs) were seeded on six‐well cell culture plates (3516, Corning) and were maintained in Endothelial Cell Growth Medium with SupplementMix (C‐22010, PromoCell, Germany). Cells were transfected with a GFP lentivirus using polybrene reagent (SC‐134220, Santa Cruz, California) following the manufacturer's instructions. Puromycin (ST551‐10, Beyotime Biotechnology, China) was used to eliminate the nontransfected cells. The GFP expression was assessed by fluorescence microscopy at 24 and 48 hours after transfection.

#### 4.2.3 *Tube formation assay*

Matrigel matrix (356234, BD Pharmingen) was diluted with Dulbecco's Modified Eagle Medium (DMEM) LOW (FBS‐free) in 1:1, 120 μL diluted Matrigel matrix was applied to the bottom of each well of a 48‐well cell culture plates (3548, Corning) and allowed to polymerize for 2 hours at 37°C. S‐MSCs or S‐MSCs‐IT was diluted with FBS‐free medium at the ratio of 1:1, 1:3, and 1:9, respectively, and 3 × 10^4^ HUVECs/well were seeded on the Matrigel matrix cultured with the diluted supernatant, with FBS‐free medium as a control. Recombinant VEGFC protein (9199‐VC‐025/CF, R&D Systems) was added at the concentration of 2500 pg/mL. After 6 hours, micrographs were taken with a cell imaging microporous plate detection system (Citation 5, Bio Tek). The number of nodes and total length were quantified by ImageJ software and Angiogenesis Analyzer plugin (https://imagej.net/macros/toolsets/Angiogenesis%20Analyzer.txt).

#### 4.2.4 *Proliferation assay*

An amount of 5 × 10^4^ HUVECs were seeded on 24‐well cell culture plate (3524, Corning). After the cells adhered to the plate, the medium was changed with the equally diluted (1:1) S‐MSCs and S‐MSCs‐IT with FBS‐free medium. Six hours later, EdU (C10310‐3, RiboBio, China) was added into the medium and incubated for 1 hour before the cells were fixed and stained.

#### 4.2.5 *Real‐time PCR analysis*

MSCs were treated with different cytokines for 24 hours and digested by trypsin. Total RNA was extracted using RNAfast 2000 (220 011, Fastagen, China) and reserve transcribed into cDNA using a PrimeScript^TM^ RT Master Mix (Perfect Real Time) (22036B, Takara, Japan) according to the manufacturer's protocol. The primers used were listed in Table 1. Reactions were performed using SYBR SELECT MASTER MIX (Thermo Fisher Scientific) in QuantStudio 6 Flex Real‐Time PCR System. The relative mRNA levels of genes were calculated by 2^−△△Ct^ method, using β‐actin as the internal control. Each averaged experimental gene expression sample was compared to the mean in the control sample, which was set to 1.

**TABLE 1:** Primers

Primer name	Sequence (5′‐3′)
Human ACTIN	TTGCCGACAGGATGCAGAAGGA AGGTGGACAGCGAGGCCAGGAT
Human VEGFA	AGGGCAGAATCATCACGAAGT AGGGTCTCGATTGGATGGCA
Human VEGFB	GAGATGTCCCTGGAAGAACACA GAGTGGGATGGGTGATGTCAG
Human VEGFC	GAGGAGCAGTTACGGTCTGTG TCCTTTCCTTAGCTGACACTTGT
Human VEGFD	TCCCATCGGTCCACTAGGTTT AGGGCTGCACTGAGTTCTTTG

#### 4.2.6 *Enzyme‐linked immunosorbent assay*

The concentration of VEGFC in S‐MSCs and S‐MSCs‐IT was measured by enzyme‐linked immunosorbent assay according to the manufacturer's description (DVEC00, R&D Systems) and 50 μL for each sample and standard was used. Absorbance (450 nm) for each sample was analyzed by a microplate reader (Cytation5, Bio Tek) and interpolated with a standard curve.

#### 4.2.7 *Animal experiments*

Six to eight‐week BALB/c female mice were purchased from Beijing Vital River Laboratory Animal Technology Co. Ltd. (Beijing, China) and maintained under a specific pathogen‐free condition. All procedures were approved and conducted under the Guideline for the Institutional Animal Care and Use Committee of Soochow University. The excisional cutaneous wound‐healing mouse model is based on the method previously described.^16^ Briefly, 3 days after adaptive feeding, two round holes with a diameter of 8 mm were dug in the back of the mice after the mice were anesthetized and then the mice were randomly divided into three groups, treated daily with medium, S‐MSCs, or S‐MSCs‐IT (each 20 μL), respectively, by topical application onto the wound bed. Wounds were then covered with a dressing film (1624W, 3M) to protect the wound from dryness and self‐grooming damage. Digital photographs of wounds were taken under an anatomic microscope (SM27457, Nikon, Japan) every day or every other day post of injury. The areas of the wound were scored by ImageJ software.

To test the role of VEGFC in angiogenesis, mice were randomly divided into four groups, and wounds were treated daily with S‐siNC‐MSCs, S‐siNC‐MSCs‐IT, S‐siVEGFC‐MSCs‐IT, or S‐siVEGFC‐MSCs‐IT‐recVEGFC, respectively. Digital photographs of wounds were taken everyday post of injury. The areas of the wound were scored by ImageJ software.

#### 4.2.8 *Wound closure measurements*

Mice were observed and digital images were taken daily. Wound areas were measured by tracing the wound margin and calculating the pixel area using image analysis software (ImageJ). The wound healing rate was calculated as follows:

$$\left[1-\left(\text{Surface of actual non}‐\mathrm{re}‐\text{epithelialized zone}\right)/\text{Surface}\;\mathrm{at}\;D0\right]*100$$

#### 4.2.9 *Hematoxylin‐eosin staining and immunohistochemistry*

Animals were euthanized and skin samples were excised, the tissues were fixed with 4% paraformaldehyde for 24 hours, then dehydrated with 70%, 75%, 85%, 95%, 100% ethyl alcohol, covered with paraffin, before sectioning and histological analysis. Blocks were cut to expose wounded tissue near the center of each wound and then cut into 4 μm thickness and stained with hematoxylin and eosin.

Number of blood vessels containing red blood cells was counted over the entire wound area using three fields per section. The thickness of the new skin evaluated as follows: The middle part of the neonatal skin wound and the two ends near the edge of the wound were, respectively, selected to measure the thickness and then calculated the average.

The tissue sections were first deparaffinized and rehydrated prior to boil in a 100°C citrate buffer water bath for 30 minutes (R20902, YUANYE, China) and then quenched of endogenous peroxidize using blocker (KIT‐9720, MXB, China) for 10 minutes before incubating with CD31(28 364, abcam, UK) antibody overnight in 4°C, to allow visualization of the immunostaining, sections were incubated with the anti‐rabbit‐biotinylated secondary antibody for 45 minutes, and then incubated with Streptaridin‐Peroxidase for 20 minutes and DAB (DAB‐2031, MXB, China) and counterstained with hematoxylin. The proportion of CD31 positive signals is calculated by ImageJ software.

#### 4.2.10 *Cytoskeleton staining*

HUVECs were cultured in S‐siNC‐MSCs, S‐siNC‐MSCs‐IT, S‐siVEGFC‐MSCs‐IT, or S‐siVEGFC‐MSCs‐IT‐recVEGFC, respectively. Twenty thousand HUVECs were seeded on Matrigel and 2 hours later, the cells were fixed with pre‐heated 4% paraformaldehyde for 5 minutes, then washed with PBS and permeated with 0.5% triton^TN^ X‐100 (V900502‐100ML, Sigma) in PBS for 3 minutes. The phalloidin (40735ES75, Yeasen, China) was used to stain the cell F‐actin based on the manufacturer's instructions and the nuclei were stained with Hoechst 33324 (H3570, Thermo Fisher Scientific). Images were taken by a laser‐scanning confocal microscope (Leica TCS SP8, Leica, Germany).

#### 4.2.11 *Immunofluorescence analysis*

The tissue sections were first deparaffinized and rehydrated prior to boil in a 100°C citrate buffer water bath for 30 minutes and then washed with PBS and permeated with 0.5% triton^TN^ X‐100 (V900502‐100ML, Sigma) in PBS followed by 3% fetal bovine serum (A602440, BBI, China) for 1 hour before incubating with primary antibodies overnight at 4°C. On the next day, sections were washed with PBS and incubated with the secondary antibodies for 1 hour at room temperature. The nuclei were stained with Hoechst 33324. The antibodies against proliferating cell nuclear antigen (PCNA) (ab29, Abcam) and Cytokeratin 14 (ab181595, Abcam) were used as primary antibodies. Secondary antibodies were Alexa 488‐conjugated‐goat anti‐rabbit IgG (ab150077, Abcam) and Alexa 647‐conjugated‐goat anti‐mouse IgG (ab150115, Abcam). Images were taken by a laser‐scanning confocal microscope (Leica TCS SP8, Leica, Germany). We analyzed the PCNA^+^ keratinocytes near the edge of the wounds and calculated the proliferation ratio.

#### 4.2.12 *Sirius red staining*

Sirius red staining kit (PSR‐1, ScyTek) was used to visualize collagen fibrils according to the manufacturer's instructions. The pictures were taken with an inverted fluorescence microscope (Ts2R, Nikon, Japan).

#### 4.2.13 *Statistical analysis*

Statistical analysis was performed using GraphPad Prism 6 software. Results of multiple observations are presented as means ± SD. Differences between two groups were assessed using unpaired Student's *t* test, for multivariate data analysis, group differences were assessed using one‐way analysis of variance with Tukey comparisons, a value of *P* < .05 was considered significant.

## CONFLICT OF INTEREST

The authors indicated no potential conflicts of interest.

## AUTHOR CONTRIBUTIONS

M.Z.: conception and design, data acquisition, analysis, writing—original draft preparation; Y. Chu: conception and design, data acquisition, analysis; Q.S.: conception and design, writing—original draft preparation; Z.Z.: conception and design; Y.L., L.C., J.C.: data acquisition; Y. Chen: administrative support; O.K.L., Y.W., G.M.: data analysis and interpretation; G.L., C.S., Y.S.: conception, design, data analysis and interpretation, supervision of study conduct and operations, writing—review and editing, final approval of manuscript.

## DATA AVAILABILITY STATEMENT

The data that support the findings of this study are available from the corresponding author upon reasonable request

## Supporting information


**APPENDIX** **S1**: Supporting Information
