# Supplementary material for: Mesenchymal stromal cells pretreated with pro‐inflammatory cytokines promote skin wound healing through VEGFC‐mediated angiogenesis
Source: Stem Cells Transl Med. 2020 Jun 13;9(10):1218–32. doi: 10.1002/sctm.19-0241 (PMC7519767; doi:10.1002/sctm.19-0241)
Supplement: Supplementary file 1 — APPENDIX S1: Supporting Information [file SCT3-9-1218-s001.zip › SCT3_12757_supplemental figures-legends.docx]

**FIG. S1 The identification of surface markers and differentiation of human umbilical cord-derived MSCs**

(A) Expression ratio of CD34, CD45, CD31, CD146, CD73, CD29, CD90, CD11b and CD105 surface markers on MSCs detected by flow cytometry. (B) Identification of MSCs through adipogenic differentiation. Scale = 100 μm. (C) Identification of MSCs through osteoplastic differentiation. Scale = 1000 μm. (D) The doubling time of MSCs. The MSCs are from the human umbilical cord (defined as SD4)

**FIG. S2** **The supernatant derived from ADSCs pre-treated with IT promotes wound closure**

Excisional wounds were treated daily with control medium, S-MSCs or S-MSCs-IT and photographs were taken. (A) Representative images of wounds are shown at the time points indicated. (B) Measurements of wound sizes at different times. Significant differences were determined by one-way ANOVA, **P* < 0.05 (n = 5-6 mice for each group and time point). Data are one independent experiment. The MSCs are from the human adipose tissue (defined as 1223^#^). Data are shown as mean ± SD.

**FIG. S3 Supernatant derived from MSCs pre-treated with IT promotes angiogenesis during skin wound healing**

(A)(C) Cutaneous wounds on day 3 and day 5 post of injury were stained with H&E and micrographs were taken. Black arrows indicate blood vessels containing red blood cells. Scale bar = 100 μm. (B)(D) The numbers of blood vessels containing red blood cells in each section were counted at the indicated time points. Results are presented as the number of blood vessels per mm^2^. Significant differences were determined by one-way ANOVA, ***P* < 0.01, *****P* < 0.0001 (*n* = 3-5 mice for each

group). Data are representative of two independent experiments. (E)(G) Representative photographs showing CD31 immunohistochemistry in

cutaneous wounds on day 4 and day 6 post of injury. Scale bar = 100 μm. (F)(H) Graph of CD31 immunohistochemistry in these groups. Results are presented as the CD31 positive area (%). Significant differences were determined by one-way ANOVA, ***P* < 0.01, ****P* < 0.001, *****P* < 0.0001 (*n* = 4-6 mice for each group). Data are one independent experiment. The MSCs are from the human umbilical cord (defined as SD4). Data are shown as mean ± SD.

**FIG. S4 Supernatant derived from another human umbilical cord-derived MSCs pre-treated with IT promotes the formation of capillary-like structures by endothelial cells in vitro.**

(A) Tube-formation assay: representative images showing tube formation of HUVECs cultured in control medium, S-MSCs or S-MSCs-IT on Matrigel. Scale bar = 500 μm. (B) Quantitative analysis of total length and (C) number of nodes for 6 h. Data are representative of three independent experiments. The MSCs are from the human umbilical cord (defined as UC506). Data are shown as mean ± SD.

**FIG. S5 Supernatant derived from ADSCs pre-treated with IT promotes the formation of capillary-like structures by endothelial cells in vitro**

(A) Tube-formation assay: representative images showing tube formation of HUVECs cultured in control medium, equally diluted (1:1) S-MSCs or S-MSCs-IT on Matrigel for 6 h. Scale bar = 1000 μm. (B) Quantitative analysis of total length. Differences were determined by one-way ANOVA, **P* < 0.05. Data are one independent experiment. The MSCs are from the human adipose tissue (defined as 1223^#^). Data are shown as mean ± SD.

**FIG. S6 IT stimulation could upregulate the expression of VEGFC in ADSCs**

(A) The mRNA expression of VEGFC in MSCs and (B) the protein concentration in MSC culture supernatant. Significance was determined by one-way ANOVA, *****P* < 0.0001. Data are representative of two independent experiments. The MSCs are from the human adipose tissue (defined as 1223^#^). Data are shown as mean ± SD.

**FIG. S7 VEGFC does not promote HUVECs proliferation and protect HUVECs from H_2_O_2_-induced apoptosis.**

(A) Proliferation of endothelial cells treated with different concentrations of VEGFC (0 ng/mL, 2 ng/mL, 10 ng/mL, 50 ng/mL, 100 ng/mL, 250 ng/mL), as determined by flow cytometry. Data are representative of two independent experiments.

(B) The protective effect of VEGFC treatment on H_2_O_2_‑induced HUVEC damage, as determined by flow cytometry. Data are representative of two independent experiments. The MSCs are from the human umbilical cord (defined as SD4).

**FIG. S8 VEGFC mediates the proliferation of keratinocytes during skin wound healing**

(A) Cutaneous wounds on day 6 post of injury were stained with PCNA and Cytokeratin 14 and micrographs were taken. Scale bar = 50 μm. (B) Quantification of the number of PCNA-positive keratinocytes. Significant differences were determined by one-way ANOVA, **P* < 0.05 (*n* = 4-6 mice for each group). Data are representative of two independent experiments. The MSCs are from the human umbilical cord (defined as SD4). Data are shown as mean ± SD.

**FIG. S9 VEGFC promotes angiogenesis during skin wound healing**

Cutaneous wounds on day 3 (A-B) and day 5 (C-D). (A)(C) Cutaneous wounds were stained with H&E and micrographs were taken. Black arrows indicate blood vessels containing red blood cells. Scale bar = 100 μm. (B)(D) The number of blood vessels containing red blood cells in each section was counted at the indicated time points. Results are presented as the number of blood vessels per mm^2^. Data are representative of two independent experiments. Significant differences were determined by one-way ANOVA, ***P* < 0.01, ****P* < 0.001 (*n* = 4-6 mice for each group). The MSCs are from the human umbilical cord (defined as SD4). Data are shown as mean ± SD.
