# Supplementary material for: Mesenchymal stromal cells pretreated with pro‐inflammatory cytokines promote skin wound healing through VEGFC‐mediated angiogenesis
Source: Stem Cells Transl Med. 2020 Jun 13;9(10):1218–32. doi: 10.1002/sctm.19-0241 (PMC7519767; doi:10.1002/sctm.19-0241)
Supplement: Supplementary file 1 — APPENDIX S1: Supporting Information [file SCT3-9-1218-s001.zip › SCT3_12757_supplemental figures-materials and methods.docx]

**SUPPLEMENTAL MATERIALS AND METHODS**

**Characterization of MSCs**

MSCs were examined for cell surface markers CD90-PE (eBioscience, USA), CD105-PE (eBioscience, USA), CD29-PE (eBioscience, USA), CD73-PE (eBioscience, USA), CD146-PE (eBioscience, USA), CD31-PE (eBioscience, USA), CD34-PE (eBioscience, USA), CD45-PE (eBioscience, USA), CD11b-PE (eBioscience, USA) and the identification for their differentiation ability of adipogenesis and osteogenesis were carried out as previously described (Fig S1). MSCs used in this study were in passages 5 to 10.

**Human adipose-derived stromal cells**

ADSCs were obtained from the adipose tissue of lipoaspirate samples based on the approved ethics protocols by the Institutional Review Board and Informed Patient Consent of the Second Affiliated Hospital of Soochow University. Adipose tissues were washed extensively with phosphate‐buffered saline (PBS) to remove debris and treated with 0.1% collagenase I in DMEM/F12 medium with 10% fetal bovine serum (FBS) for 1 h at 37°C with gentle agitation. The digested samples were centrifuged at 400 g for 5 min to separate adipocytes from stromal vascular fraction. The cell pellet containing the stromal fraction was resuspended in DMEM/F12 supplemented with 10% FBS, 100 U/mL penicillin/streptomycin solution and 10 ng/mL bFGF and cultured in 10 cm dishes at 37°C with 5% CO2. Nonadherent cells were removed after 24 h, and adherent cells were maintained with medium replenishment every 3 days.

**Proliferation assay**

10 × 10^4^ HUVECs were seeded on 12-well cell culture plate (3516, Corning, USA), 12 h later, recombinant VEGFC protein were added into the medium and incu­bated at 37˚C for 24 h at various concentrations. Subsequently, EdU (C0081S, BeyoClick™ EdU-555, [Beyotime Biotechnology](http://www.baidu.com/link?url=EJ8ZA1vdkgMxXtDY_BTbM0MbwJlM_aBTFmT13YoWCxpQ8N3KSGXJCtEn0XG5BhG6)) was added into the medium and incubated for 2 h. The proliferation was measured by flow cytometry according to the manufacturer’s instructions.

**Apoptosis analysis:**

Briefly, 10 × 10^4^ HUVECs were seeded on 12-well cell culture plate (3516, Corning, USA), 12 h later, The cells were treated with various concentrations of VEGFC or H_2_O_2_ for 24 h, then the cell apoptosis was evaluated by flow cytometry using an Annexin Ⅴ-APC and 7AAD-PerCP-Cy5.5-A according to the manufacturer’s instructions.

The early apoptosis was measured by quantifying the population of Annexin Ⅴ^+^7AAD^-^ cells for 10,000 events, the late apoptosis or necrosis was measured by quantifying the population of Annexin Ⅴ^+^7AAD^+^ cells for 10,000 events.
