# Supplementary material for: Mesenchymal stromal cells pretreated with pro‐inflammatory cytokines promote skin wound healing through VEGFC‐mediated angiogenesis
Source: Stem Cells Transl Med. 2020 Jun 13;9(10):1218–32. doi: 10.1002/sctm.19-0241 (PMC7519767; doi:10.1002/sctm.19-0241)
Supplement: Supplementary file 1 — APPENDIX S1: Supporting Information [file SCT3-9-1218-s001.zip › SCT3_12757_supplemental.pdf]

SUPPLEMENTAL MATERIALS AND METHODS

Characterization of MSCs

MSCs were examined for cell surface markers CD90-PE (eBioscience, USA), CD105-PE (eBioscience, USA), CD29-PE (eBioscience, USA), CD73-PE (eBioscience, USA), CD146-PE (eBioscience, USA), CD31-PE (eBioscience, USA), CD34-PE (eBioscience, USA), CD45-PE (eBioscience, USA), CD11b-PE (eBioscience, USA) and the identification for their differentiation ability of adipogenesis and osteogenesis were carried out as previously described (Fig S1). MSCs used in this study were in passages 5 to 10.

Human adipose-derived stromal cells

ADSCs were obtained from the adipose tissue of lipoaspirate samples based on the approved ethics protocols by the Institutional Review Board and Informed Patient Consent of the Second Affiliated Hospital of Soochow University. Adipose tissues were washed extensively with phosphate-buffered saline (PBS) to remove debris and treated with 0.1% collagenase I in DMEM/F12 medium with 10% fetal bovine serum (FBS) for 1 h at 37°C with gentle agitation. The digested samples were centrifuged at 400 g for 5 min to separate adipocytes from stromal vascular fraction. The cell pellet containing the stromal fraction was resuspended in DMEM/F12 supplemented with 10% FBS, 100 U/mL penicillin/streptomycin solution and 10 ng/mL bFGF and cultured in 10 cm dishes at 37°C with 5% CO2. Nonadherent cells were removed after 24 h, and adherent cells were maintained with medium replenishment every 3 days.

Proliferation assay

10×10^4 HUVECs were seeded on 12-well cell culture plate (3516, Corning, USA), 12 h later, recombinant VEGFC protein were added into the medium and incubated at 37˚C for 24 h at various concentrations. Subsequently, EdU (C0081S, BeyoClick™ EdU-555, Beyotime Biotechnology) was added into the medium and incubated for 2 h. The proliferation was measured by flow cytometry according to the manufacturer's instructions.

Apoptosis analysis:

Briefly, 10×10^4 HUVECs were seeded on 12-well cell culture plate (3516, Corning, USA), 12 h later, The cells were treated with various concentrations of VEGFC or H_2O_2 for 24 h, then the cell apoptosis was evaluated by flow cytometry using an Annexin V-APC and 7AAD-PerCP-Cy5.5-A according to the manufacturer's instructions.

The early apoptosis was measured by quantifying the population of Annexin V^+7AAD^- cells for 10,000 events, the late apoptosis or necrosis was measured by quantifying the population of Annexin V^+7AAD^+ cells for 10,000 events.

SUPPLEMENTAL FIGURE LEGENDS

FIG. S1 The identification of surface markers and differentiation of human umbilical cord-derived MSCs

(A) Expression ratio of CD34, CD45, CD31, CD146, CD73, CD29, CD90, CD11b and CD105 surface markers on MSCs detected by flow cytometry. (B) Identification of MSCs through adipogenic differentiation. Scale = 100 μm. (C) Identification of MSCs through osteoplastic differentiation. Scale = 1000 μm. (D) The doubling time of MSCs. The MSCs are from the human umbilical cord (defined as SD4)

FIG. S2 The supernatant derived from ADSCs pre-treated with IT promotes wound closure

Excisional wounds were treated daily with control medium, S-MSCs or S-MSCs-IT and photographs were taken. (A) Representative images of wounds are shown at the time points indicated. (B) Measurements of wound sizes at different times. Significant differences were determined by one-way ANOVA, $*P < 0.05$ (n = 5-6 mice for each group and time point). Data are one independent experiment. The MSCs are from the human adipose tissue (defined as $1223^{\#}$). Data are shown as mean ± SD.

FIG. S3 Supernatant derived from MSCs pre-treated with IT promotes angiogenesis during skin wound healing

(A)(C) Cutaneous wounds on day 3 and day 5 post of injury were stained with H&E and micrographs were taken. Black arrows indicate blood vessels containing red blood cells. Scale bar = 100 μm. (B)(D) The numbers of blood vessels containing red blood cells in each section were counted at the indicated time points. Results are

presented as the number of blood vessels per mm^2. Significant differences were determined by one-way ANOVA, $**P < 0.01$, $****P < 0.0001$ (n = 3-5 mice for each group). Data are representative of two independent experiments. (E)(G) Representative photographs showing CD31 immunohistochemistry in cutaneous wounds on day 4 and day 6 post of injury. Scale bar = 100 μm. (F)(H) Graph of CD31 immunohistochemistry in these groups. Results are presented as the CD31 positive area (%). Significant differences were determined by one-way ANOVA, $**P < 0.01$, $***P < 0.001$, $****P < 0.0001$ (n = 4-6 mice for each group). Data are one independent experiment. The MSCs are from the human umbilical cord (defined as SD4). Data are shown as mean ± SD.

FIG. S4 Supernatant derived from another human umbilical cord-derived MSCs pre-treated with IT promotes the formation of capillary-like structures by endothelial cells in vitro.

(A) Tube-formation assay: representative images showing tube formation of HUVECs cultured in control medium, S-MSCs or S-MSCs-IT on Matrigel. Scale bar = 500 μm. (B) Quantitative analysis of total length and (C) number of nodes for 6 h. Data are representative of three independent experiments. The MSCs are from the human umbilical cord (defined as UC506). Data are shown as mean ± SD.

FIG. S5 Supernatant derived from ADSCs pre-treated with IT promotes the formation of capillary-like structures by endothelial cells in vitro

(A) Tube-formation assay: representative images showing tube formation of HUVECs cultured in control medium, equally diluted (1:1) S-MSCs or S-MSCs-IT on Matrigel

for 6 h. Scale bar = 1000 μm. (B) Quantitative analysis of total length. Differences were determined by one-way ANOVA, $*P < 0.05$. Data are one independent experiment. The MSCs are from the human adipose tissue (defined as 1223$^{\#}$). Data are shown as mean ± SD.

FIG. S6 IT stimulation could upregulate the expression of VEGFC in ADSCs

(A) The mRNA expression of VEGFC in MSCs and (B) the protein concentration in MSC culture supernatant. Significance was determined by one-way ANOVA, $****P < 0.0001$. Data are representative of two independent experiments. The MSCs are from the human adipose tissue (defined as 1223$^{\#}$). Data are shown as mean ± SD.

FIG. S7 VEGFC does not promote HUVECs proliferation and protect HUVECs from H_2O_2-induced apoptosis.

(A) Proliferation of endothelial cells treated with different concentrations of VEGFC (0 ng/mL, 2 ng/mL, 10 ng/mL, 50 ng/mL, 100 ng/mL, 250 ng/mL), as determined by flow cytometry. Data are representative of two independent experiments.

(B) The protective effect of VEGFC treatment on H_2O_2-induced HUVEC damage, as determined by flow cytometry. Data are representative of two independent experiments. The MSCs are from the human umbilical cord (defined as SD4).

FIG. S8 VEGFC mediates the proliferation of keratinocytes during skin wound healing

(A) Cutaneous wounds on day 6 post of injury were stained with PCNA and Cytokeratin 14 and micrographs were taken. Scale bar = 50 μm. (B) Quantification of the number of PCNA-positive keratinocytes. Significant differences were determined

by one-way ANOVA, $*P < 0.05$ ($n = 4$-6 mice for each group). Data are representative of two independent experiments. The MSCs are from the human umbilical cord (defined as SD4). Data are shown as mean ± SD.

FIG. S9 VEGFC promotes angiogenesis during skin wound healing

Cutaneous wounds on day 3 (A-B) and day 5 (C-D). (A)(C) Cutaneous wounds were stained with H&E and micrographs were taken. Black arrows indicate blood vessels containing red blood cells. Scale bar = 100 μm. (B)(D) The number of blood vessels containing red blood cells in each section was counted at the indicated time points. Results are presented as the number of blood vessels per mm^2. Data are representative of two independent experiments. Significant differences were determined by one-way ANOVA, $**P < 0.01$, $***P < 0.001$ ($n = 4$-6 mice for each group). The MSCs are from the human umbilical cord (defined as SD4). Data are shown as mean ± SD.

Figure S1

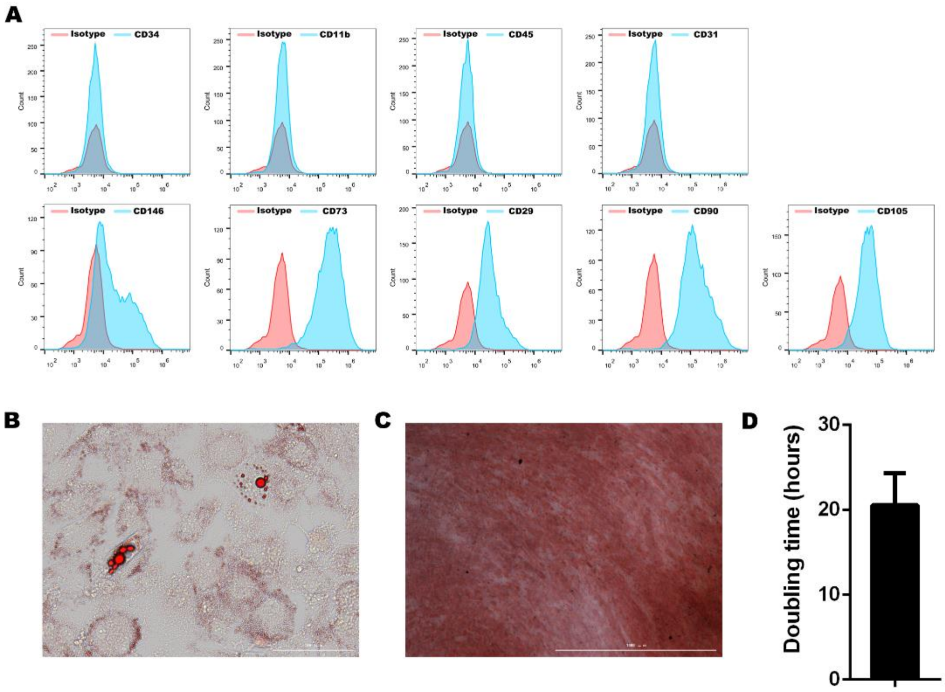

A
Isotype
CD34
CD11b
CD45
CD31
CD146
CD73
CD29
CD90
CD105
Count
B
C
D
Doubling time (hours)
30
20
10
0

Figure S2

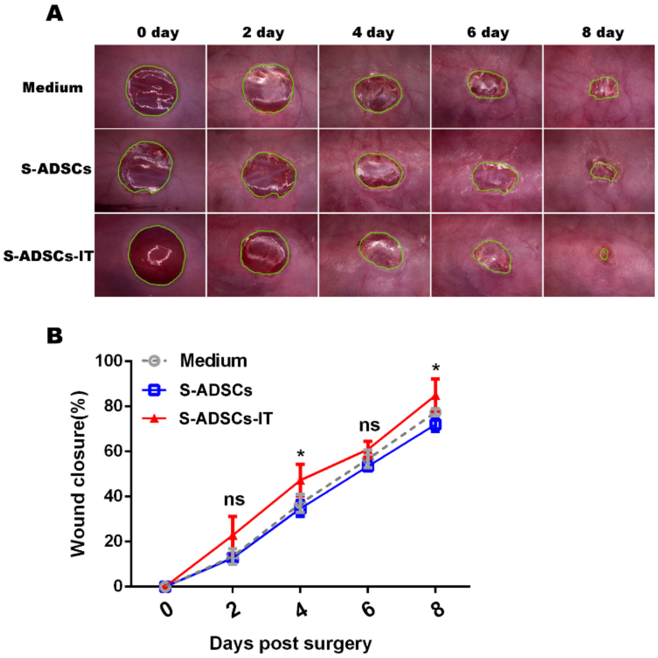
A
0 day
2 day
4 day
6 day
8 day
Medium
S-ADSCs
S-ADSCs-IT
B
Medium
S-ADSCs
S-ADSCs-IT
Wound closure(%)
100
80
60
40
20
0
ns
*
ns
*
0
2
4
6
8
Days post surgery

Figure S3

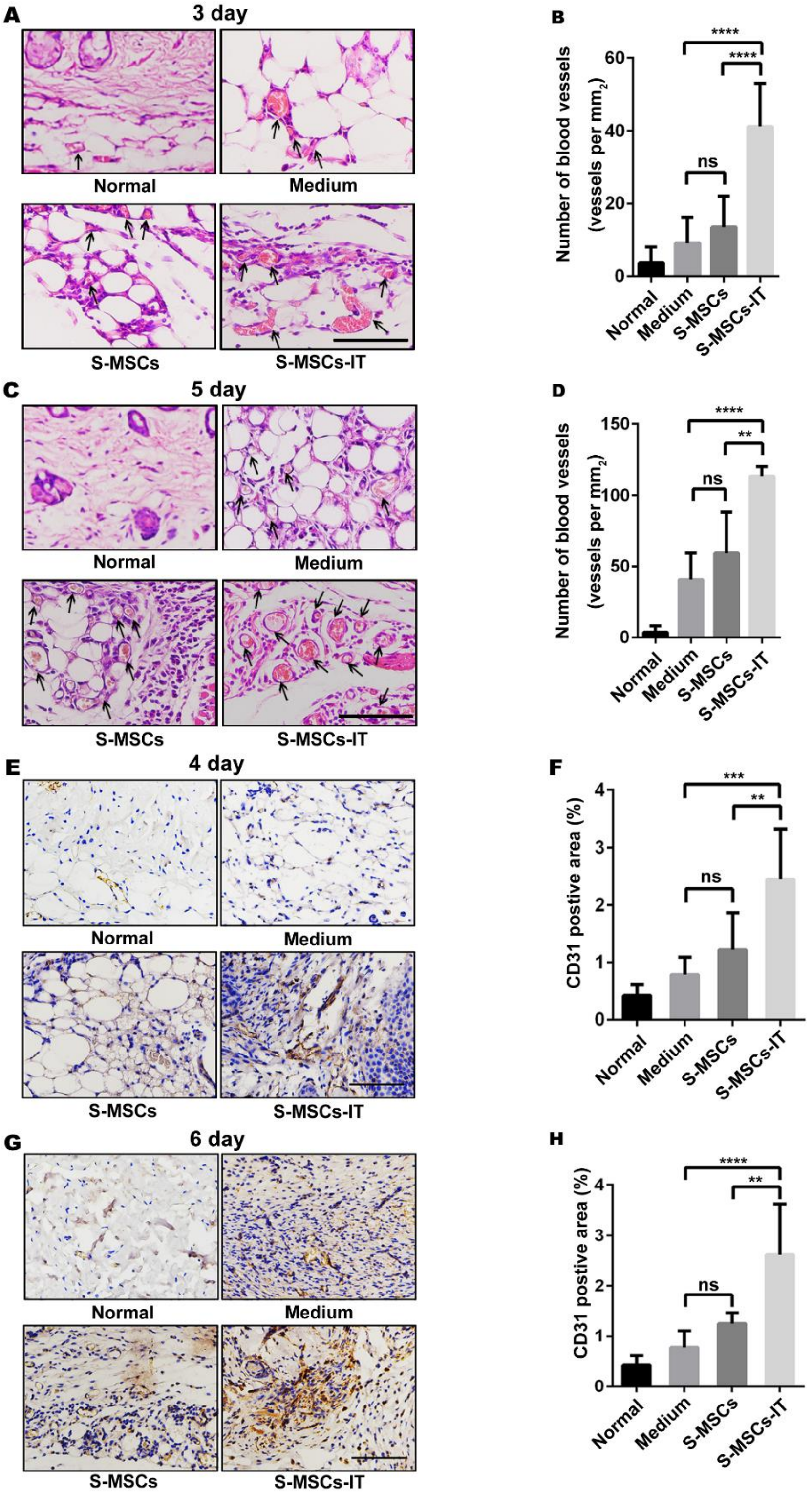

A
3 day
Normal
Medium
S-MSCs
S-MSCs-IT
B
Number of blood vessels (vessels per mm2)
60
40
20
0
****
****
ns
Normal
Medium
S-MSCs
S-MSCs-IT
C
5 day
Normal
Medium
S-MSCs
S-MSCs-IT
D
Number of blood vessels (vessels per mm2)
150
100
50
0
****
**
ns
Normal
Medium
S-MSCs
S-MSCs-IT
E
4 day
Normal
Medium
S-MSCs
S-MSCs-IT
F
CD31 postive area (%)
4
3
2
1
0
***
**
ns
Normal
Medium
S-MSCs
S-MSCs-IT
G
6 day
Normal
Medium
S-MSCs
S-MSCs-IT
H
CD31 postive area (%)
4
3
2
1
0
****
**
ns
Normal
Medium
S-MSCs
S-MSCs-IT

Figure S4

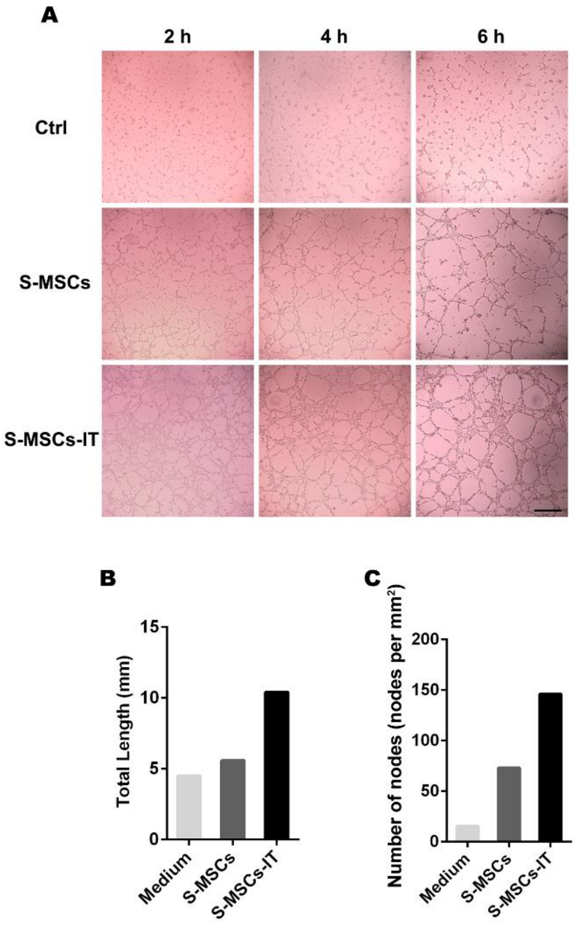

A
2 h
4 h
6 h
Ctrl
S-MSCs
S-MSCs-IT
B
Total Length (mm)
15
10
5
0
Medium
S-MSCs
S-MSCs-IT
C
Number of nodes (nodes per mm²)
200
150
100
50
0
Medium
S-MSCs
S-MSCs-IT

Figure S5

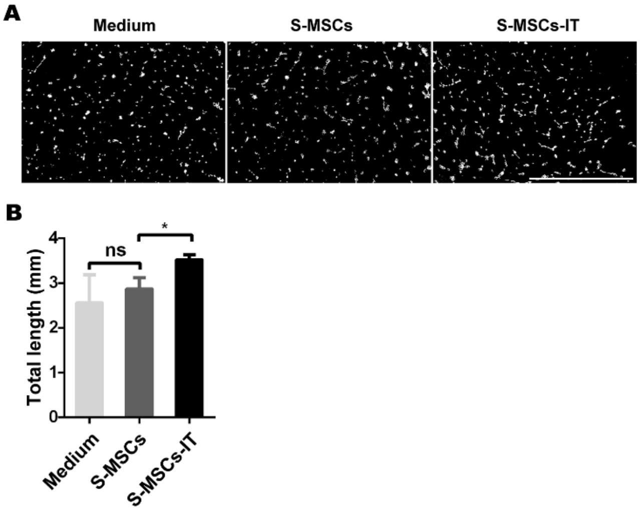
A
Medium
S-MSCs
S-MSCs-IT
B
Total length (mm)
4
3
2
1
0
ns
*
Medium
S-MSCs
S-MSCs-IT

Figure S6

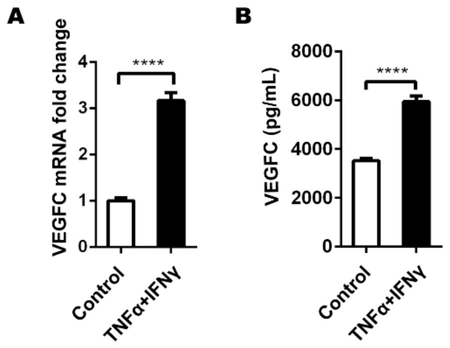
A
VEGFC mRNA fold change
4
3
2
1
0
****
Control
TNFα+IFNγ
B
VEGFC (pg/mL)
8000
6000
4000
2000
0
****
Control
TNFα+IFNγ

Figure S7

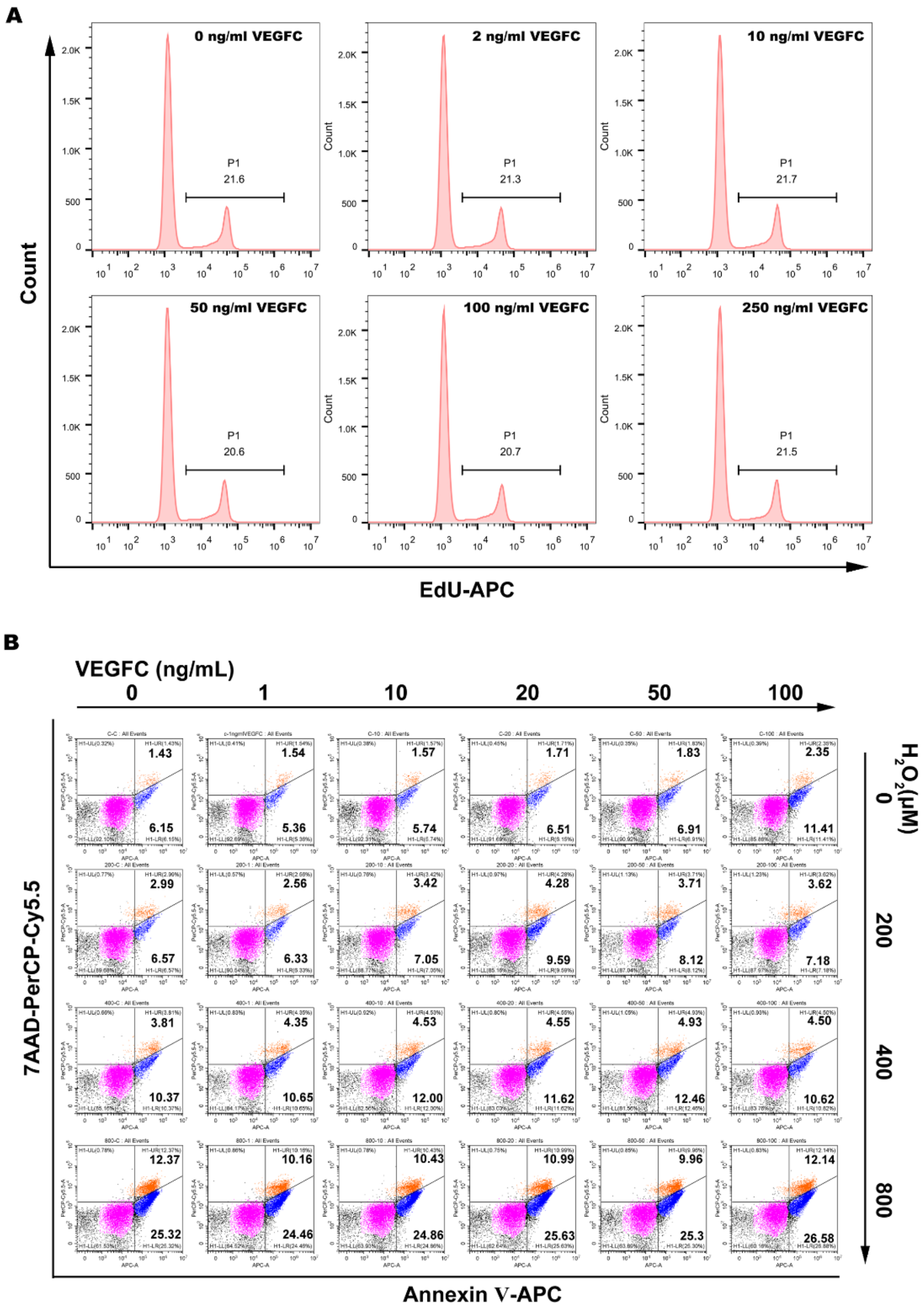

A
0 ng/ml VEGFC
2 ng/ml VEGFC
10 ng/ml VEGFC
50 ng/ml VEGFC
100 ng/ml VEGFC
250 ng/ml VEGFC
P1
21.6
21.3
21.7
20.6
20.7
21.5
Count
EdU-APC
B
VEGFC (ng/mL)
0
1
10
20
50
100
H_2O_2(μM)
0
200
400
800
7AAD-PerCP-Cy5.5
Annexin V-APC
1.43
1.54
1.57
1.71
1.83
2.35
6.15
5.36
5.74
6.51
6.91
11.41
2.99
2.56
3.42
4.28
3.71
3.62
6.57
6.33
7.05
9.59
8.12
7.18
3.81
4.35
4.53
4.55
4.93
4.50
10.37
10.65
12.00
11.62
12.46
10.62
12.37
10.16
10.43
10.99
9.96
12.14
25.32
24.46
24.86
25.63
25.3
26.58

Figure S8

A

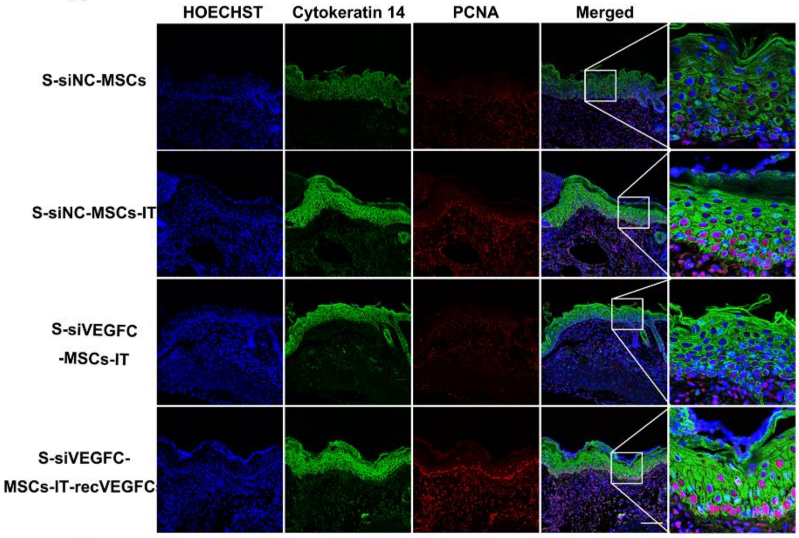
HOECHST
Cytokeratin 14
PCNA
Merged
S-siNC-MSCs
S-siNC-MSCs-IT
S-siVEGFC
-MSCs-IT
S-siVEGFC-
MSCs-IT-recVEGFC

B

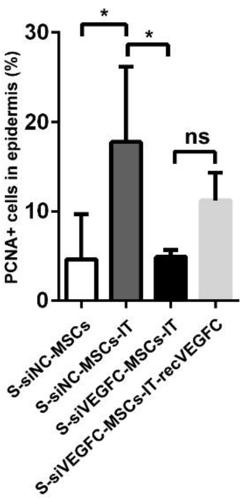
PCNA+ cells in epidermis (%)
30
20
10
0
*
*
ns
S-siNC-MSCs
S-siNC-MSCs-IT
S-siVEGFC-MSCs-IT
S-siVEGFC-MSCs-IT-recVEGFC

Figure S9

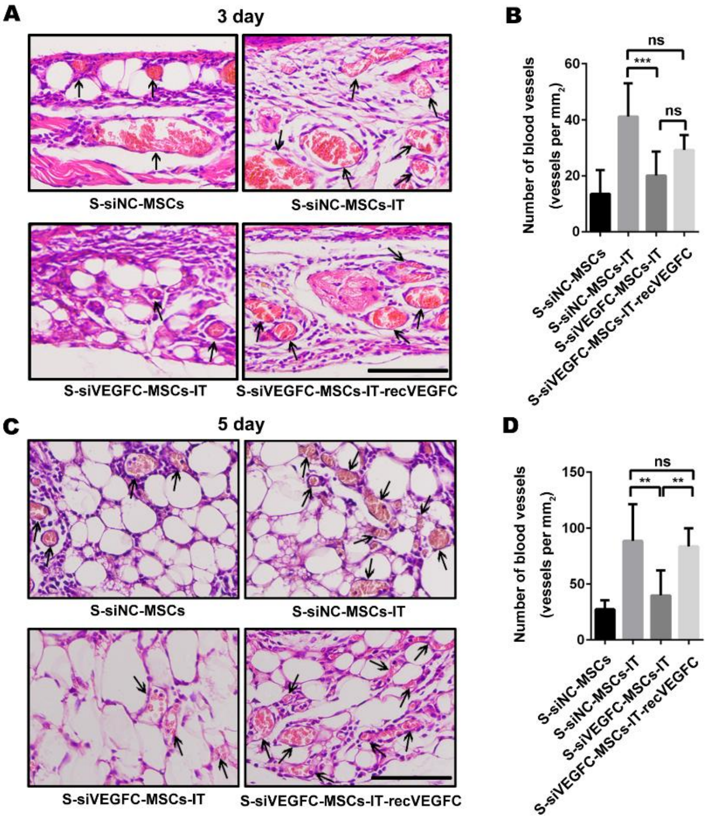

A
3 day
S-siNC-MSCs
S-siNC-MSCs-IT
S-siVEGFC-MSCs-IT
S-siVEGFC-MSCs-IT-recVEGFC
B
Number of blood vessels (vessels per mm2)
0
20
40
60
ns
***
ns
S-siNC-MSCs
S-siNC-MSCs-IT
S-siVEGFC-MSCs-IT
S-siVEGFC-MSCs-IT-recVEGFC
C
5 day
S-siNC-MSCs
S-siNC-MSCs-IT
S-siVEGFC-MSCs-IT
S-siVEGFC-MSCs-IT-recVEGFC
D
Number of blood vessels (vessels per mm2)
0
50
100
150
ns
**
**
S-siNC-MSCs
S-siNC-MSCs-IT
S-siVEGFC-MSCs-IT
S-siVEGFC-MSCs-IT-recVEGFC
