# Supplementary figures and images for: Mesenchymal stromal cells pretreated with pro‐inflammatory cytokines promote skin wound healing through VEGFC‐mediated angiogenesis
Source: Stem Cells Transl Med. 2020 Jun 13;9(10):1218–32. doi: 10.1002/sctm.19-0241 (PMC7519767; doi:10.1002/sctm.19-0241)

**Figure S1**

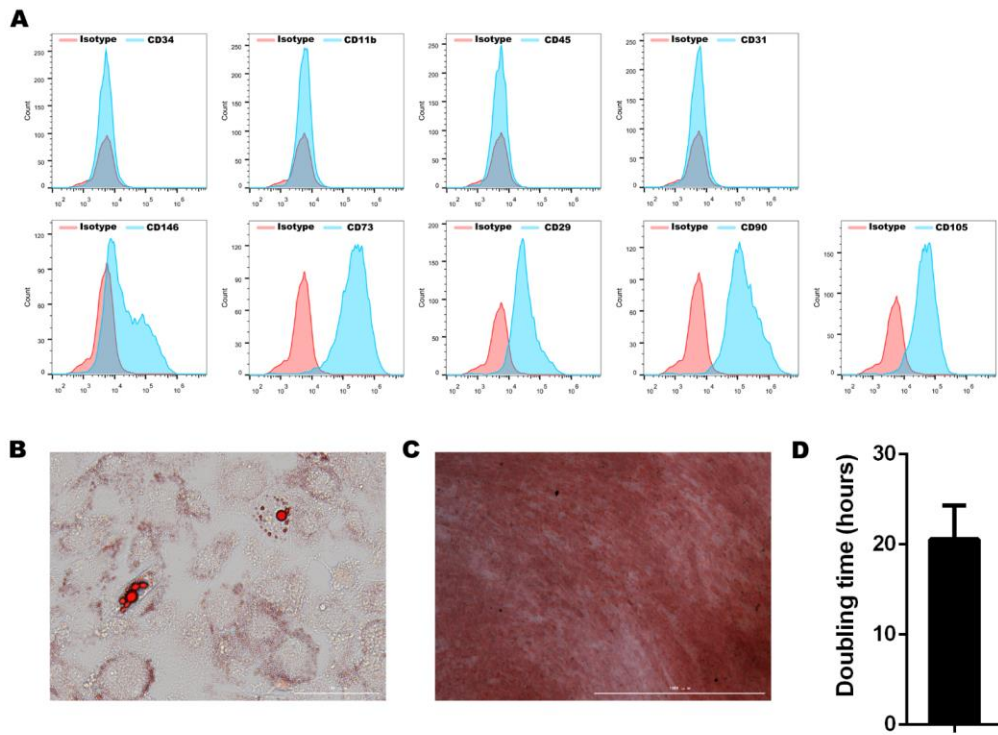

Figure S2

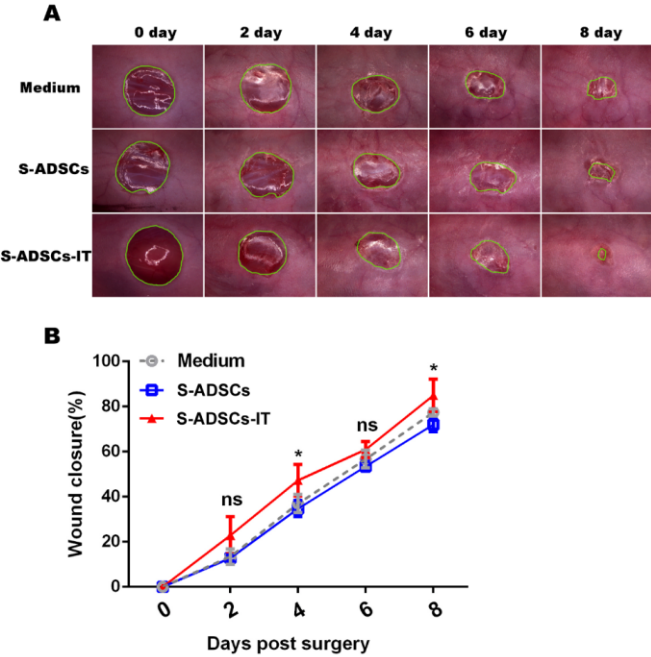

**Figure S3**

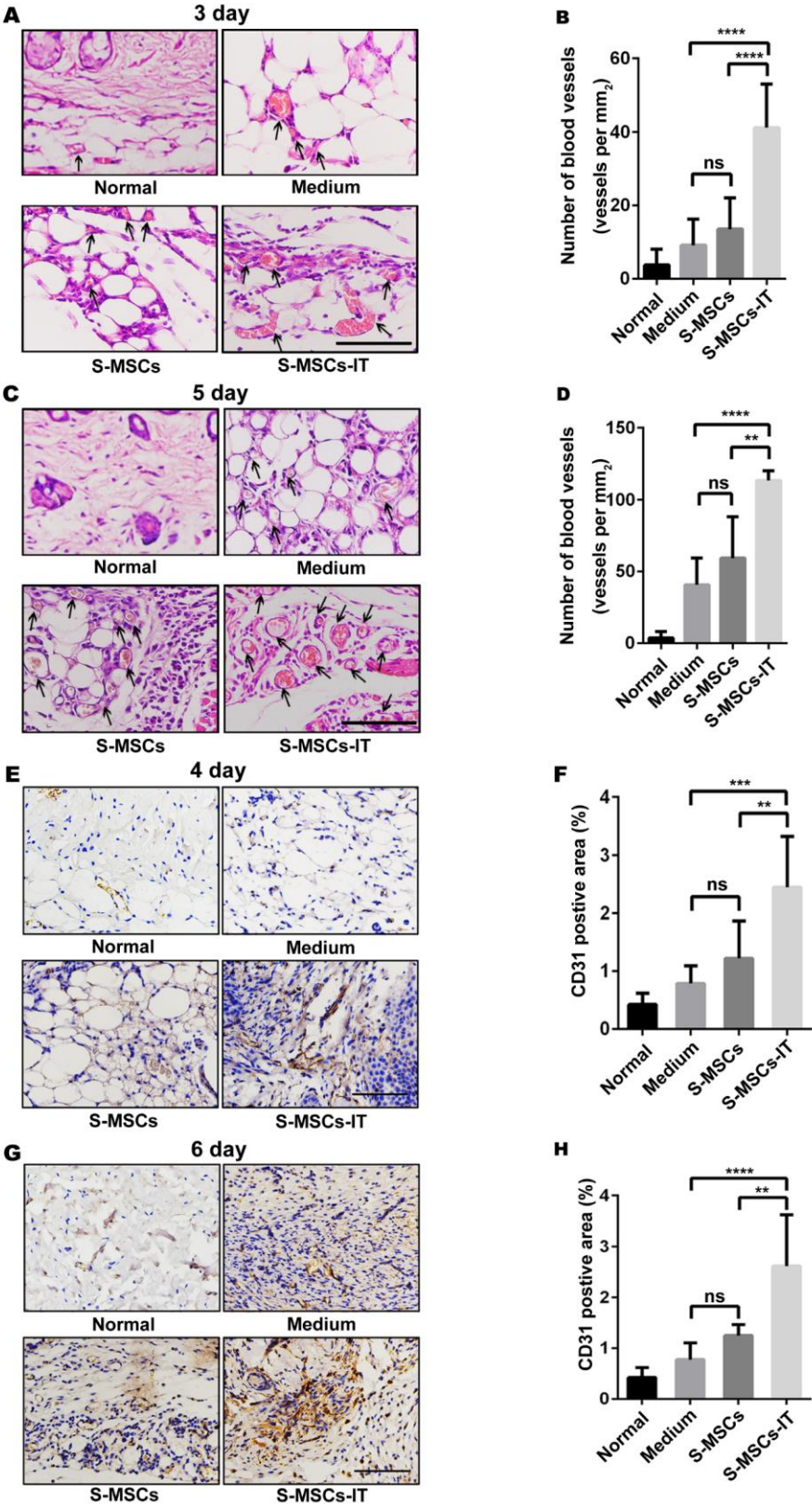

**Figure S4**

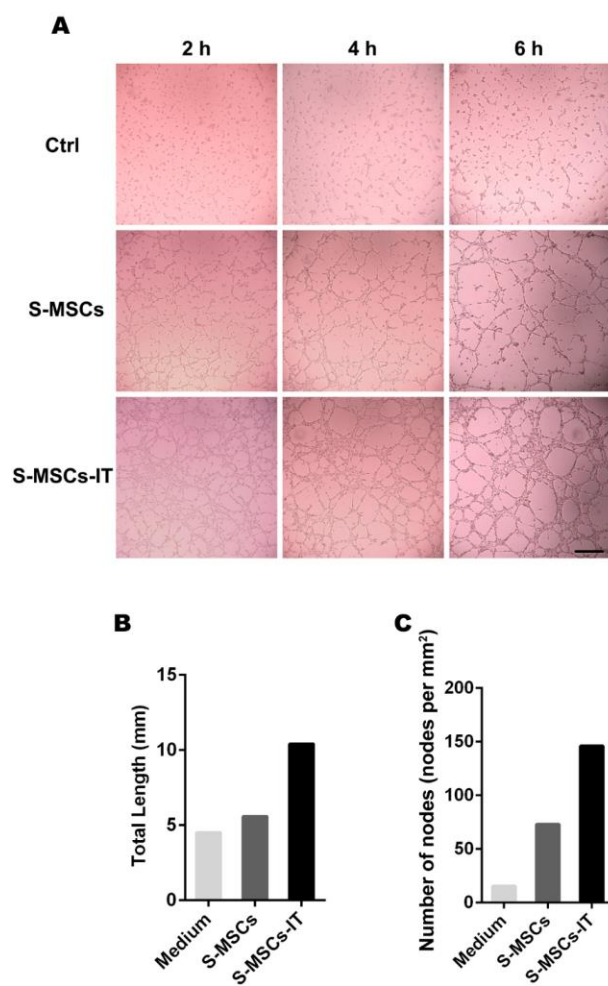

**Figure S5**

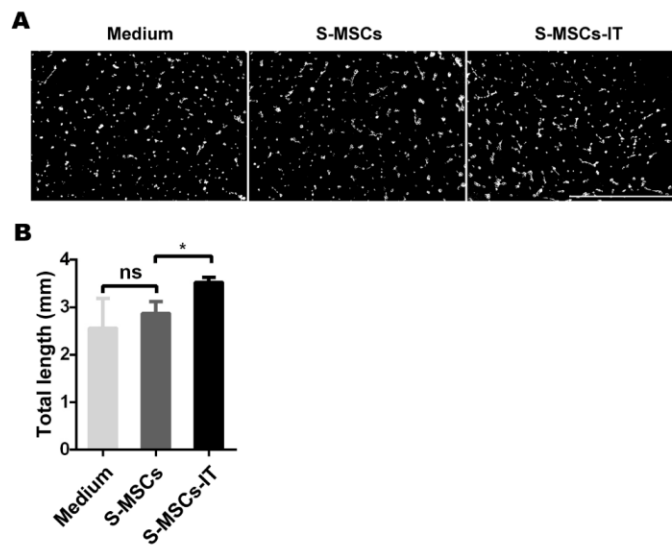

**Figure S6**

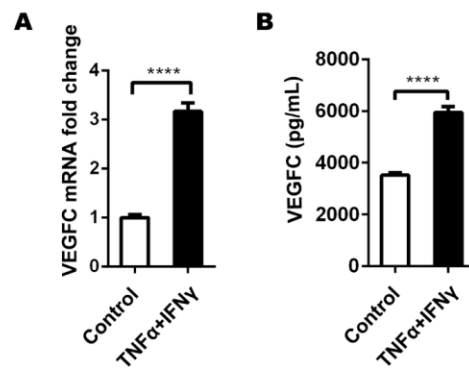

**Figure S7**

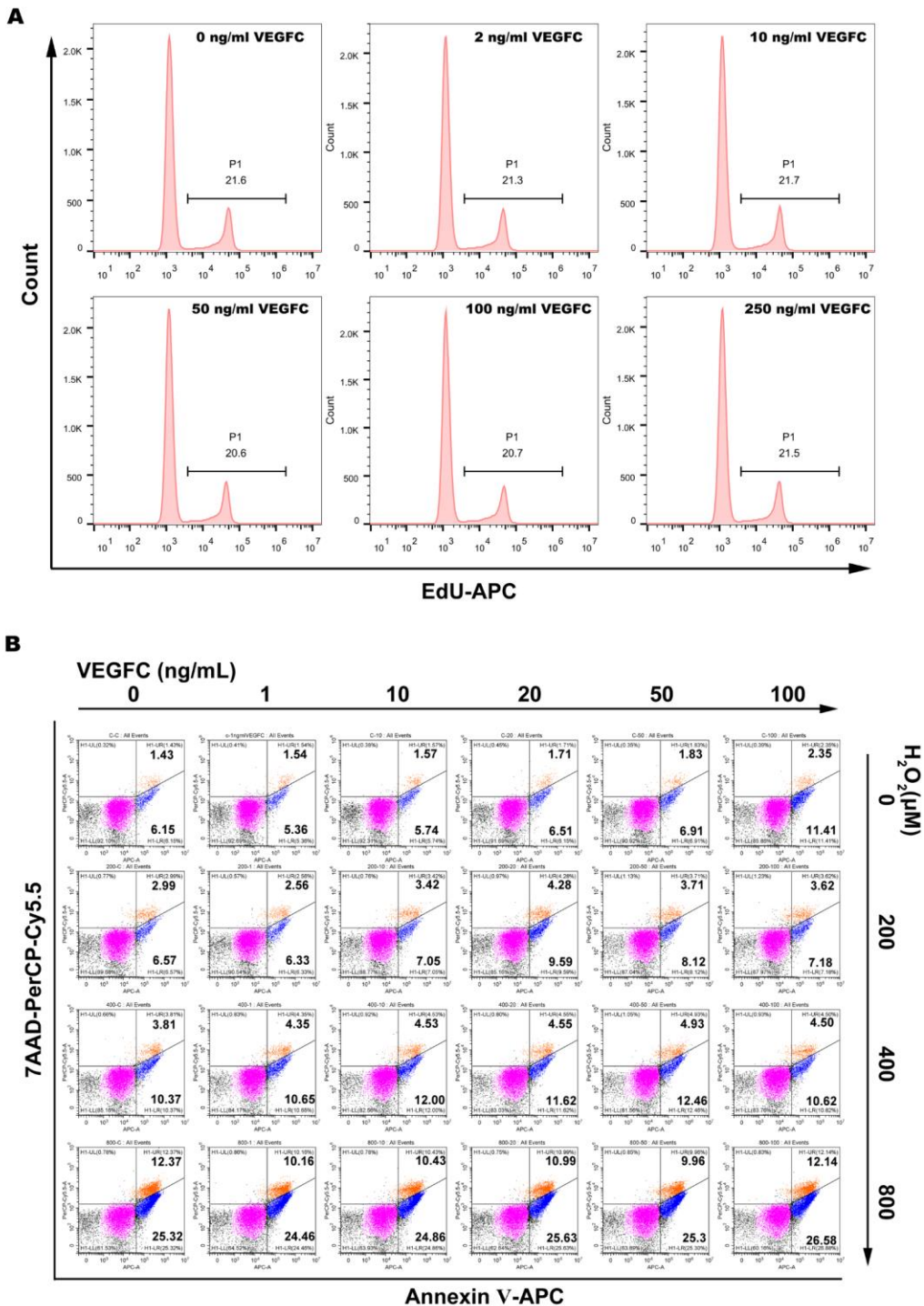

**Figure S8**

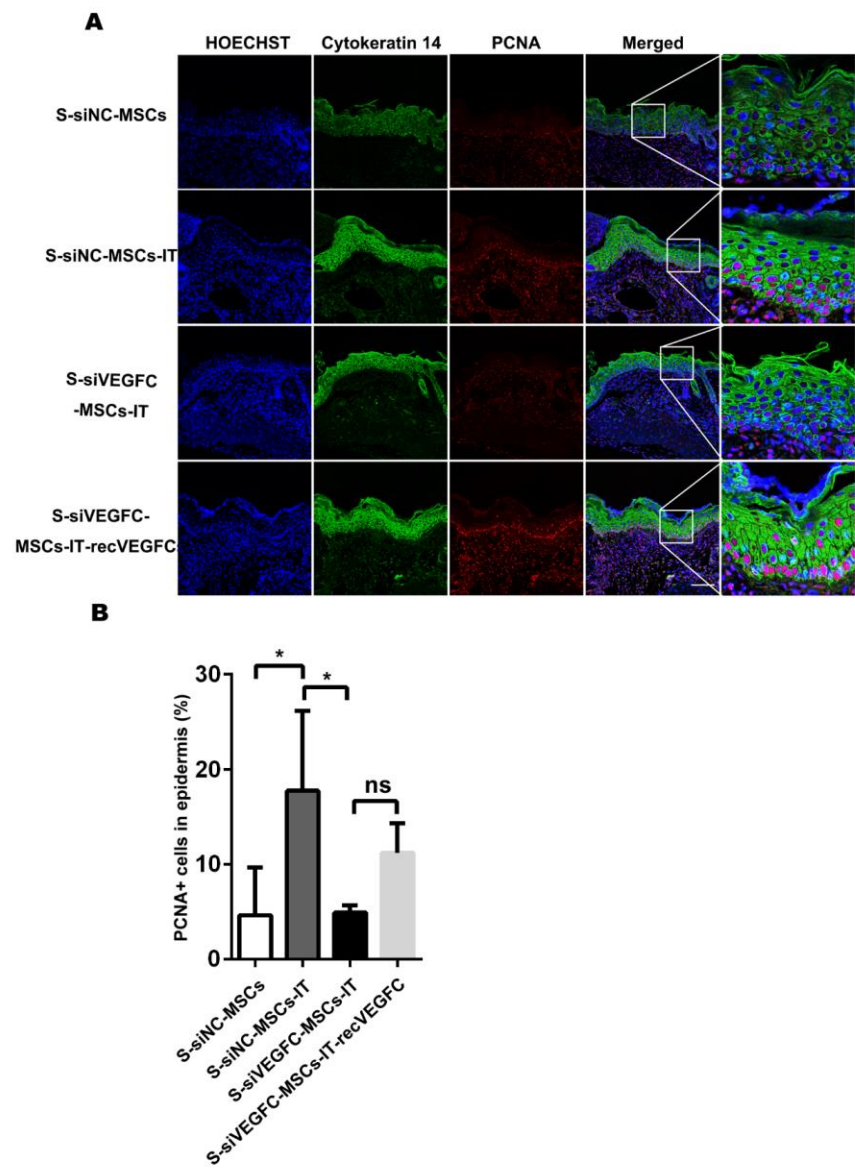

**Figure S9**

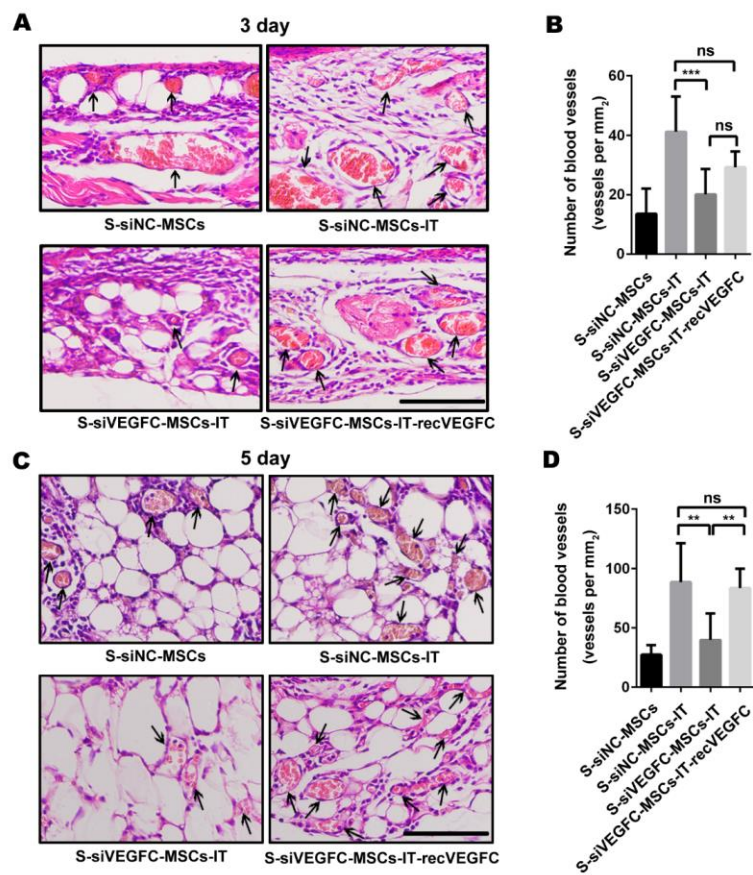

Supplement: Supplementary file 1 — APPENDIX S1: Supporting Information [file SCT3-9-1218-s001.zip › SCT3_12757_supplemental figures.pdf]
